# Challenges and Lessons Learned in Managing Web-Based Survey Fraud for the Garnering Effective Outreach and Research in Georgia for Impact Alliance–Community Engagement Alliance Survey Administrations

**DOI:** 10.2196/51786

**Published:** 2024-12-24

**Authors:** Leslie S Craig, Christina L Evans, Brittany D Taylor, Jace Patterson, Kaleb Whitfield, Mekhi Hill, Michelle Nwagwu, Mohamed Mubasher, Robert A Bednarczyk, Gail G McCray, Cheryl L R Gaddis, Natasha Taylor, Emily Thompson, Ursula Douglas, Saundra K Latimer, Sedessie G Spivey, Tabia Henry Akintobi, Rakale Collins Quarells

**Affiliations:** 1 Strategic Consulting Solutions, Christ Church, Barbados; 2 Department of Family Medicine, Morehouse School of Medicine, Atlanta, GA, United States; 3 Health Strategies Division, American Heart Association, Atlanta, GA, United States; 4 Department of Modern Language, Ohio University, Athens, OH, United States; 5 Georgia Health Policy Center, Georgia State University, Atlanta, GA, United States; 6 Community Health and Preventive Medicine, Morehouse School of Medicine, Atlanta, GA, United States; 7 Hubert Department of Global Health, Rollins School of Public Health, Emory University, Atlanta, GA, United States; 8 Department of Public Health, Mercer University College of Health Professions, Atlanta, GA, United States; 9 Georgia Watch, Atlanta, GA, United States; 10 Community Friendship Inc, Atlanta, GA, United States; 11 Sprout Consulting Group, Atlanta, GA, United States; 12 Health Assessment and Promotion Department Division of Community Health, DeKalb Public Health, Decatur, GA, United States

**Keywords:** web-based survey research, data quality, data integrity, COVID-19, Georgia, data collection, scientists, integrity, transparency, public health, deception, disinformation, survey fraud, legitimate data

## Abstract

**Background:**

Convenience, privacy, and cost-effectiveness associated with web-based data collection have facilitated the recent expansion of web-based survey research. Importantly, however, practical benefits of web-based survey research, to scientists and participants alike, are being overshadowed by the dramatic rise in suspicious and fraudulent survey submissions. Misinformation associated with survey fraud compromises data quality and data integrity with important implications for scientific conclusions, clinical practice, and social benefit. Transparency in reporting on methods used to prevent and manage suspicious and fraudulent submissions is key to protecting the veracity of web-based survey data; yet, there is limited discussion on the use of antideception strategies during all phases of survey research to detect and eliminate low-quality and fraudulent responses.

**Objective:**

This study aims to contribute to an evolving evidence base on data integrity threats associated with web-based survey research by describing study design strategies and antideception tools used during the web-based administration of the Garnering Effective Outreach and Research in Georgia for Impact Alliance–Community Engagement Alliance (GEORGIA CEAL) Against COVID-19 Disparities project surveys.

**Methods:**

GEORGIA CEAL was established in response to the COVID-19 pandemic and the need for rapid, yet, valid, community-informed, and community-owned research to guide targeted responses to a dynamic, public health crisis. GEORGIA CEAL Surveys I (April 2021 to June 2021) and II (November 2021 to January 2022) received institutional review board approval from the Morehouse School of Medicine and adhered to the CHERRIES (Checklist for Reporting Results of Internet E-Surveys).

**Results:**

A total of 4934 and 4905 submissions were received for Surveys I and II, respectively. A small proportion of surveys (Survey I: n=1336, 27.1% and Survey II: n=1024, 20.9%) were excluded due to participant ineligibility, while larger proportions (Survey I: n=1516, 42.1%; Survey II: n=1423, 36.7%) were flagged and removed due to suspicious activity; 2082 (42.2%) and 2458 (50.1%) of GEORGIA CEAL Surveys I and II, respectively, were retained for analysis.

**Conclusions:**

Suspicious activity during GEORGIA CEAL Survey I administration prompted the inclusion of additional security tools during Survey II design and administration (eg, hidden questions, Completely Automated Public Turing Test to Tell Computers and Humans Apart verification, and security questions), which proved useful in managing and detecting fraud and resulted in a higher retention rate across survey waves. By thorough discussion of experiences, lessons learned, and future directions for web-based survey research, this study outlines challenges and best practices for designing and implementing a robust defense against survey fraud. Finally, we argue that, in addition to greater transparency and discussion, community stakeholders need to be intentionally and mindfully engaged, via approaches grounded in community-based participatory research, around the potential for research to enable scientific discoveries in order to accelerate investment in quality, legitimate survey data.

## Introduction

Practical benefits of web-based survey research—including convenience to researchers and participants, relatively low study costs, increased privacy, and subsequent lower potential for socially desirable responses, in addition to ease of data entry and analysis—are well described in the literature [1-5]. Between 2020 and 2022, during the height of the COVID-19 pandemic, when quarantines and social distancing recommendations created a surge in the need for remote data collection, a simultaneous explosion in web-based survey research took place [1,2,6-8]. Rapid, convenient, and cost-effective data collection facilitated by the use of anonymous web-based surveys maintains the appeal of web-based survey research among clinical researchers and social scientists [2,9]. Web-based research, despite these advantages, is not without its challenges.

Suspicious responses, fraudulent participation, and internet robot (ie, bot) submissions present substantial threats to sample validity in web-based survey research [3,5,6,10,11]. Specifically, web-based surveys may be accessed multiple times by potential respondents, for legitimate (eg, the individual is unsure if survey data were captured on the first attempt) or illegitimate (eg, the individual maliciously seeks additional compensation) reasons, or by nonhuman, automated bots capable of completing web-based forms randomly and systematically [6,7,12]. Whether the result of unintentional, albeit careless, completion of multiple surveys or the purposeful misrepresentation of oneself [1], misinformation associated with web-based survey fraud compromises data quality and integrity with important implications for scientific conclusions, clinical practice, and social benefit [2,7].

The dramatically increasing prevalence of fraudulent responses in web surveys, coupled with increasing reliance on web-based platforms for research, underscores the need to protect the veracity of web-based survey data [13]. This is further evidenced by active discourse within the scientific community, describing preventative strategies (eg, instrument design and web-based platform selection) and exclusionary measures (eg, data cleaning and analytic checks) that may prove useful in combating web-based survey fraud [1,3,9]. Given increasing bot sophistication, an evolving technological landscape, and ongoing challenges, however, no gold standard approach yet exists, and researchers continue to call for increased discussion and transparency in reporting on real-world experiences, best practice recommendations, and lessons learned in preventing and managing suspicious and fraudulent submissions [1,7,9,10,14-17].

Garnering Effective Outreach and Research in Georgia for Impact Alliance–Community Engagement Alliance (GEORGIA CEAL) Against COVID-19 Disparities is a project funded by the National Institutes of Health and co-led by the National Heart, Lung, and Blood Institute and the National Institute on Minority Health and Health Disparities, since September 2020, as part of a national, statewide initiative to conduct innovative, community-responsive, research and outreach to understand and address vaccine hesitancy, misinformation and mistrust toward acceptance, confidence, and uptake of COVID-19 vaccinations among racial or ethnic and rural communities disproportionately affected by the pandemic. This paper outlines approaches and lessons learned during GEORGIA CEAL’s web-based survey research efforts, with an aim to inform the prevention, detection, and exclusion of suspicious and fraudulent records during all phases of web-based survey research. Building on published evidence and study findings, we describe our experiences with web-based survey administration across 2 survey waves, including context-specific considerations and data quality approaches as well as methodological strengths and potential limitations of outlined strategies, in an effort to add to the scientific discussion on data integrity threats associated with web-based survey research and contribute to an evolving evidence base describing tools that may be implemented (predata collection, during data collection, and postdata collection) to mount a robust defense against suspicious and fraudulent survey submissions.

## Methods

### Study Design and Population

GEORGIA CEAL surveys were jointly developed by the National CEAL Assessment and Evaluation Workgroup, which defined optional and core items reflecting important themes and social determinants, and the GEORGIA CEAL Community Coalition Board (CCB), which refined and culturally adapted survey items to ensure relevance to Georgia communities. The conceptual development of GEORGIA CEAL surveys is described in further detail elsewhere [18]. To minimize participant burden, surveys were designed to be completed within 15‐20 minutes with the use of skip logic to condition access to specific questions based on prior responses. GEORGIA CEAL Surveys I and II contained 86 and 138 main questions, respectively, in either a single-question or matrix format. Participants were not allowed to change their answers via a back button feature.

Given the disproportionate burden of COVID-19 and the goal of understanding its impact among hardest hit populations, GEORGIA CEAL survey eligibility criteria were restricted to adults (ie, 18 years of age or older) of Black or African American race or Hispanic or Latino or a Latinx ethnicity. There were 2 rounds of GEORGIA CEAL surveys. For GEORGIA CEAL Survey I (April 2021 to June 2021), respondents also needed to be a resident in 1 of 19 prespecified Georgia counties; given the changing pandemic landscape, the geographic inclusion criteria were expanded for GEORGIA CEAL Survey II, administered between November 2021 and January 2022, to include 34 prespecified Georgia counties. Counties were chosen based on the proportion of Black or African American or Latinx county residents, low COVID-19 testing rates, high COVID-19 infection rates, low COVID-19 vaccination rates, and the county’s Social Vulnerability Index developed by the Centers for Disease Control and Prevention [19].

### Participant Recruitment and Data Collection

The GEORGIA CEAL CCB, comprised of 35 members representing diverse organizations from across the state, was leveraged to engage and recruit Georgian people from the priority populations. In brief, an email outlining the study purpose, eligibility criteria, and participation incentive was shared with CCB members following a discussion during the regular monthly CCB meeting. The email included a prewritten recruitment message, with content in English and Spanish, survey links, and recruitment flyers (paper and electronic) for broad distribution within the CCB’s contact lists or listservs and social media networks (Multimedia Appendix 1).

Data were predominantly collected on the web via Qualtrics, although some in-person data collection at GEORGIA CEAL community events, using iPads, also took place. Respondents were first asked to complete a series of screening questions to determine eligibility before being directed to a detailed consent form. Those who gave informed consent were then directed to a voluntary, open survey to complete a series of questions on demographics, social determinants of health, and COVID-19 beliefs and experiences (MultimediaAppendices 2  3). For Survey I, 2 different surveys, one in English and another in Spanish, were created, while, for Survey II, a translation feature was used that enabled participants to toggle and complete surveys in their preferred language.

### Statistical Analysis

Data were exported from the Qualtrics platform and analyzed in Stata (StataCorp) to examine suspicious activity and fraudulent responses. Results are discussed below, including details on the administration of both survey waves and iterative changes to the study protocol, consent script, and survey.

### Ethical Considerations

The study followed the CHERRIES (Checklist for Reporting Results of Internet E-Surveys) guidelines to ensure completeness in reporting study methods and results (Checklist 1) [20]. All study procedures were approved by the Morehouse School of Medicine Institutional Review Board (IRB) and completed in accordance with institutional guidelines (1664429). Participant privacy and confidentiality were protected via adherence to a strict data handling protocol; survey data with personal identifiers were downloaded onto encrypted servers, deidentified using unique ID numbers, and stored as password-protected files. Eligible individuals who consented to and completed the survey received a US $25 e-gift card.

## Results

Within the first 2 weeks of the GEORGIA CEAL Survey I going live, suspicious behavior, namely substantially high numbers of submitted surveys, was detected. For example, while 25 survey responses were submitted on April 9th, 2568 responses were received on April 21st, representing a daily total higher than the planned sample size (n=2004) for the full survey data collection. This high volume of responses received was cause for concern and alerted the GEORGIA CEAL team to the need for the development and implementation of a systematic and vigilant monitoring process.

As a first step, study protocol and consent form amendments were submitted to the IRB, including explicit language that persons would not be compensated if discovered to have completed duplicate or fraudulent surveys. GEORGIA CEAL initially informed respondents that “We ask you to give your name, email, or address to get a US $25 gift card as a thank you for your time. You do not have to provide us with your name, email, or address to join, but we will not be able to send the gift card without it,” but later revised the informed consent form to add a statement that “All surveys will be authenticated prior to the US $25 gift card being sent out.” In addition, surveys submitted on the Qualtrics platform were examined daily and, where suspicious behavior was detected (eg, dramatic increases in the number of survey responses recorded during a short time frame and several survey responses submitted within minutes of each other), access to the survey was paused, for approximately 1 to 3 days, to ascertain the quality and integrity of survey responses and determine needed action.

This combination of daily survey monitoring, coupled with temporary pauses when indicated, was essential for the timely detection of unusual survey response patterns and the determination of whether sample size goals had been met. Once a survey was temporarily paused, those in the process of responding to questionnaire items were allowed to complete the survey but others were unable to start a new survey. During the temporary pauses, team members validated zip codes by reviewing free-text entries and ensuring that they corresponded not only to one of the eligible Georgia counties but also to the specific county that had been selected on the survey. In addition, names, email addresses, and survey completion times were reviewed, and unusual responses flagged as suspicious. Based on the authenticated surveys, county quotas were then updated to enable eligible individuals from remaining counties to gain access to the survey. Once the process was completed, survey data collection resumed, and new respondents were able to access and complete surveys until county recruitment goals were met.

**Figure 1.**
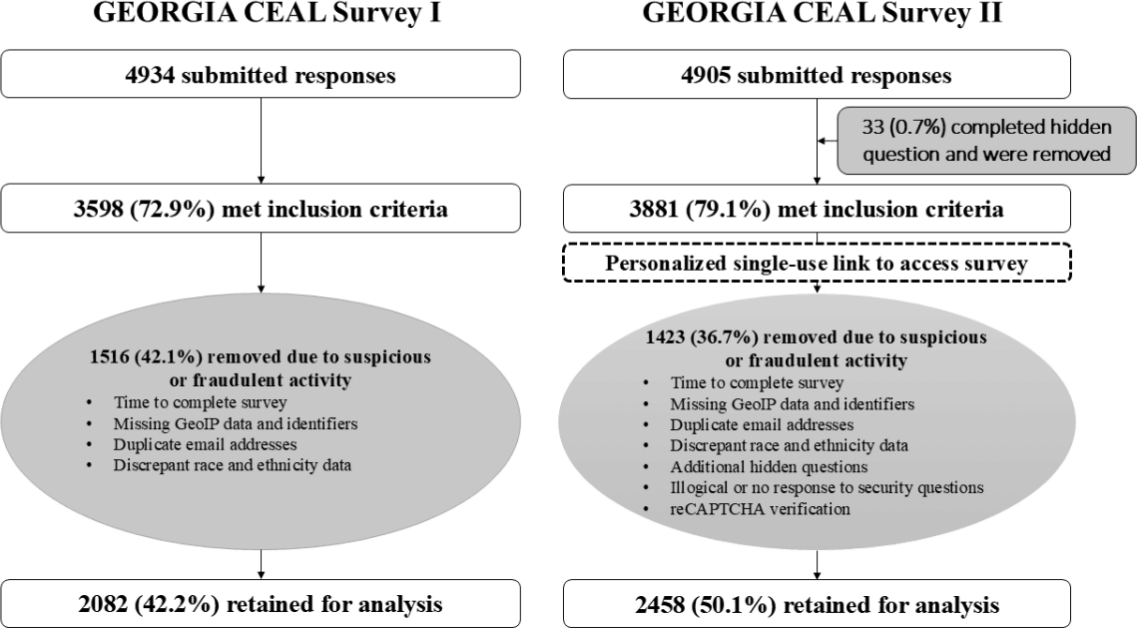
Diagram showing GEORGIA CEAL Surveys I and II’s process of elimination of suspicious or fraudulent records. CAPTCHA: Completely Automated Public Turing Test to Tell Computers and Humans Apart; GEORGIA CEAL: Garnering Effective Outreach and Research in Georgia for Impact Alliance–Community Engagement Alliance.

During data analysis, several parameters were used to exclude low-quality and fraudulent submissions, starting with inclusion criteria that were assessed at the beginning of the survey (Figure 1). Of the 4934 records received, 1336 (27.1%) were ineligible based on county, race or ethnicity, age, and informed consent status and were removed.

An additional 1516 (30.7%) records were removed following a review of flags used to authenticate survey responses, including timestamps, geolocation (ie, latitude and longitude) data, missing or duplicate identifiers, and incongruent race and ethnicity data responses in the screening form compared to the questionnaire, that were implemented in a stepwise fashion. Specifically, nearly one-third of the remaining submissions (n=1133, 31.5%) reflected survey completion in less than 10 minutes and were excluded from the analytic dataset. An additional 64 responses were missing geolocation data and, upon closer inspection, excluded from analysis due to incompleteness and missing all identifiers (ie, first name, last name, and email address). A further 201 submissions where respondents failed to specify an email address in addition to any first or last name were removed from the analysis. Conversely, the 46 submissions with duplicate email addresses were individually reviewed, as responses often had the same first and last name and the same survey submission date, and, in these instances, the decision was made to keep only the first submitted entry (ie, 23 records were excluded). Finally, 95 submissions with discrepant race and ethnicity data in the screening form compared to the questionnaire were removed, leaving 2082 GEORGIA CEAL Survey I responses for analysis.

Having been primed to the challenges of web-based survey fraud, several features available on the Qualtrics platform were incorporated into GEORGIA CEAL Survey II to flag low-quality or suspicious survey responses. These included having potential respondents first complete a Completely Automated Public Turing Test to Tell Computers and Humans Apart (CAPTCHA) before proceeding to answer a series of screening questions to determine eligibility to participate. Screening questions were preceded by a hidden question with embedded JavaScript (ie, “What number comes after 59?”) that was visible only to bots and used as another step in establishing human identity. Individuals who did not see (and thus did not answer) this hidden question were directed to the study screening questions, and those who reportedly met survey inclusion criteria then proceeded to a detailed study informed consent page. Those who gave written informed consent were sent single-use personalized links to access and complete the survey.

Similar to GEORGIA CEAL Survey I, and as an added effort to identify fraudulent users attempting to falsely pass screening questions and qualify for participation, questions on race and age were repeated within the main questionnaire to further facilitate consistency checks. Additional hidden questions with JavaScript, as well as security questions asking respondents to identify visual cues, were embedded throughout the survey as added checks. At the end of the survey, respondents were asked to complete open-ended questions providing some combination of personal identifiers including their first and last name, email address, and physical address. Collection of survey metadata, including time and date stamps, IP address, and geolocation, was also used to determine potentially fraudulent responses.

The steps used to identify and exclude suspicious and fraudulent submissions from the 4905 GEORGIA CEAL Survey II responses received are depicted in Figure 1. Following the removal of 33 (0.7%) records who responded to the hidden question, additional 991 (20.2%) responses were excluded for failing to meet geographic (n=130, 2.7%) and racial or ethnic (n=725, 14.8%) inclusion criteria and not providing written (n=136, 2.8%) informed consent. Other measures used to flag and exclude suspicious and fraudulent submissions included answering hidden questions, taking less than 10 minutes to complete the survey, providing no response or an illogical response on security questions (eg, using Chinese characters such as 蓝色汽车 or Latin words such as Architecto Quibusdam), missing geolocation data as well as all personal identifiers, missing or suspicious email addresses, and discrepant race or ethnicity data. Bot detection software on the Qualtrics platform generates a reCAPTCHA score ranging from 0.0 to 1.0 to flag responses that are more likely to be from a bot than a human. Qualtrics further advises that scores greater than or equal to 0.5 are likely to be human; accordingly, responses with a score lesser than 0.5 or missing were excluded from the analysis. As with GEORGIA CEAL Survey I, only the first submitted entry was retained in the case of duplicate email addresses, particularly as these respondents often provided identical names and submission dates. A final total of 2458 responses were retained for analysis.

## Discussion

### Principal Findings

Our experiences with web-based survey data collection highlight the extensiveness of low-quality and fraudulent data, emphasizing the need for antifraud measures to protect data quality and promote rigor in research. Specifically, the smaller proportion of surveys retained for analyses in GEORGIA CEAL Survey I (2082/4934, 42.2%) compared to GEORGIA CEAL Survey II (2458/4905, 50.1%) suggests that the use of additional security tools (eg, reCAPTCHA verification, single-use survey access links, and hidden questions) was better in managing and detecting survey fraud. Continuous and adaptive monitoring, at all stages of the research process, were also integral to handling suspicious and fraudulent submissions received during the GEORGIA CEAL survey administration. Additional strategies included IRB resubmissions to modify study document language around incentive distribution, daily monitoring of survey responses and county quotas to ensure that population targets were being met, and rigorous data cleaning and analytic plans, which ensured data quality and integrity.

**Figure 2.**
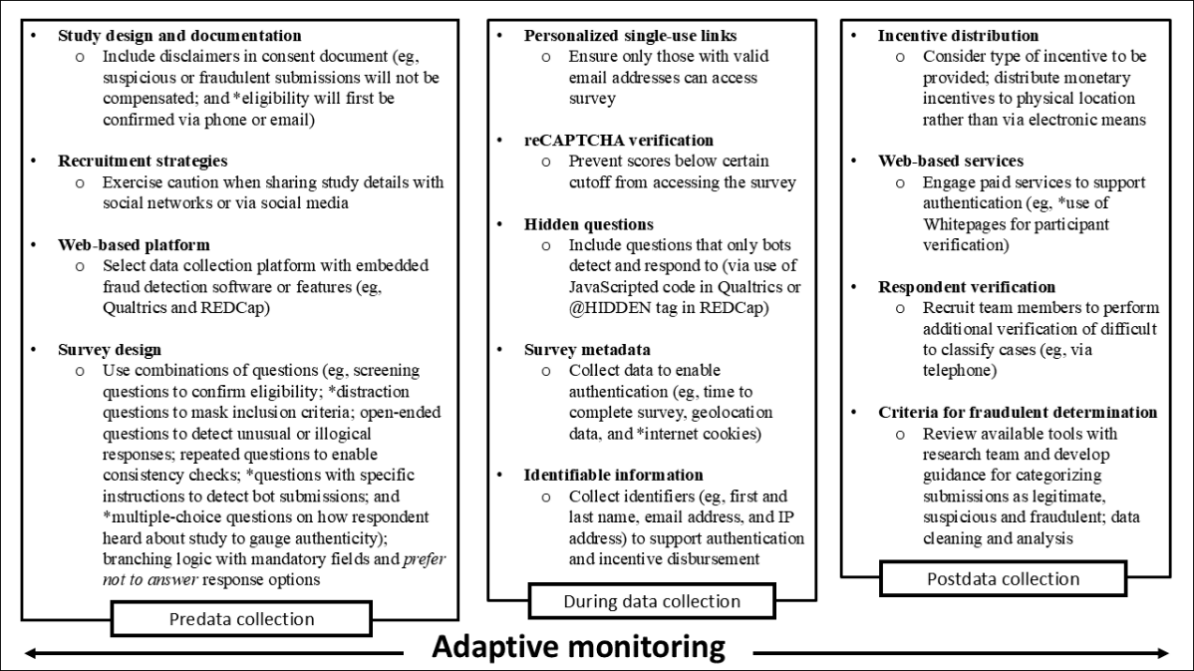
Summary of potential tools to defend against suspicious and fraudulent submissions. CAPTCHA: Completely Automated Public Turing Test to Tell Computers and Humans Apart; REDCap: Research Electronic Data Capture. * indicates strategies that were not used in GEORGIA CEAL web-based survey administration.

Transparency in reporting on methods used to protect against, detect, and remove fraudulent survey responses is also key to clarifying and addressing issues of low-quality and fraudulent data in web-based survey research. Figure 2 outlines important strategies used at all stages of the GEORGIA CEAL research process (ie, predata collection, data collection, and postdata collection) and highlights additional tools described throughout the literature that may further defend against suspicious and fraudulent submissions. Strategies marked by an asterisk denote approaches that were not used in GEORGIA CEAL web-based survey administration. Further elaboration on these approaches, via discussion of lessons learned from GEORGIA CEAL web-based survey administration (provided below), may be useful in the planning and execution of web-based research projects, by raising awareness of available survey technology and software tools, and by presenting real-world experiences to highlight best practices and caution researchers to potential challenges.

### Lessons Learned From GEORGIA CEAL Web-Based Survey Experiences

#### Predata Collection

During study conceptualization and planning, it is imperative that researchers consider the inclusion of explicit statements within study documents, and consent forms noting that suspicious or fraudulent submissions will not be compensated [2,12]. If study resources allow for individual follow-up and response authentication, this aspect of study monitoring may also be incorporated into the study design to caution against fraud and alert prospective participants that they may be contacted via telephone or email to confirm eligibility and authenticity [2].

Participant recruitment is another critical consideration in study design, with implications for the timely, informed, and equitable enrollment of a socially, diverse study sample that is representative of the priority population and does not reflect bias [21,22]. GEORGIA CEAL was able to capitalize on its CCB network’s influence during all phases of its research to raise program awareness, engage prospective participants, and support information dissemination. Yet, our experiences suggest the need for selective and intentional information-sharing when engaging social networks for the purposes of web-based recruitment. Specifically, given the ubiquity of web-based survey fraud, caution should be exercised when sharing study information, including minimizing the publication of study-specific details (eg, eligibility criteria and incentive amounts) across social media channels to keep fraudsters less aware of what responses to select and reduce malicious motivations to participate [2,23]. These measures are new considerations to balance intentional trustworthiness among minoritized and marginalized communities frequently underrepresented in public health research and practice studies while ensuring reliability and rigor in a new era of web-based survey administration.

In advance of survey design and data collection, researchers should also determine the web-based survey platform that will be used for survey administration and familiarize themselves with available fraud detection features. Two commonly used platforms for survey design and distribution, REDCap (Research Electronic Data Capture; Vanderbilt University) [24,25] and Qualtrics, allow users to embed security measures within surveys to detect bots and fraudulent activity (eg, reCAPTCHA technology; Google). Additional features, such as the multiple language translation feature in Qualtrics, permit the instant, seamless translation of surveys into the language of choice, minimize the financial, personnel, and time resources otherwise needed to translate study documents, create separate surveys, ensure that the anonymized link shared (if one is being used) is truly associated with the preferred-language survey, and download and merge separate datasets hosting responses to each preferred-language survey. This feature also preserves the agency of the research team by enabling the editing of survey items after translation, as is needed, to ensure relevance to the geographic, community, and cultural context. Cultural or contextual relevance and content validity can further be strengthened via collaboration with experts who are well-poised to evaluate the alignment of the questions with the study’s goals as well as the clarity and ease of language of survey questions to potential survey responders, in addition to pilot-testing.

Finally, antifraud protections may be incorporated into survey design via the purposeful inclusion of a variety of question types and formats (eg, screening questions, open-ended questions, repeated questions, and security questions). Presenting screening questions initially, prior to allowing access to the full survey, enables confirmation of whether participants meet study eligibility criteria and, when combined with distraction questions (ie, random questions that are not used to determine eligibility and may not even be related to the study purpose), further helps with making inclusion criteria less clear to those who may attempt to access the survey multiple times with malicious intent [10]. While multiple-choice response options support easier coding and data analysis, the inclusion of some open-ended questions facilitates the detection of unusual or illogical responses [2]. Including pairs of questions is useful for examining discrepant responses and may be integrated into the survey via a mix of open-ended and closed-ended questions (eg, asking a respondent to select from a predefined list the month and year of birth early in the survey and then repeating this age question later on by asking that the age in years be written out) to strengthen consistency checks [2,10,22]. The use of security questions asking respondents to follow specific instructions or describe a visible prompt and the use of hidden questions that are detectable only to bots (ie, in Qualtrics via embedded JavaScript) may aid fraudulent survey and bot submission detection [11]. Some authors additionally recommend the use of study-specific, multiple-choice questions (eg, asking how respondents heard about the study) to assist with data verification [23]. Given that data missingness is an important data quality dimension [26,27], specific design approaches may be incorporated into web-based survey programming to enable completeness, including skip logic, field validation programming (ie, permitting only specific number, date, or other formats), and requiring a response to all relevant questions. When making pertinent questions mandatory, the inclusion of “prefer not to answer” response options is crucial not only to minimize nonresponse but also to respect a respondent’s right to refuse to answer any question. For open-ended questions, enabling only specific formats (eg, requiring a telephone number field to include 7 digits) helps minimize data entry errors while, for questions with predefined categories (eg, eligible counties), specifying options in a drop-down menu (with an “other” category as is needed) helps ease data cleaning burdens.

#### Data Collection

Distribution of personal, single-use survey links—which only permit those who have a valid email address to access the survey instrument—appeared to be among the most effective antifraud prevention features used in GEORGIA CEAL Survey II compared to GEORGIA CEAL Survey I. This is in keeping with reports from the literature, where researchers similarly opted to privately email unique, one-time use links to web-based surveys to protect data quality, following evidence of fraudulent responses in web-based recruitment efforts [28]. Recognizing potential challenges in collecting data from older individuals who may not have email addresses or access to web technology and ensuring survey access and representation across all age groups, GEORGIA CEAL survey protocols were designed to allow in-person outreach via staff-administered surveys. In these instances, the participant’s phone number may have been used for the email address field (eg, 7775551234@gaceal.edu), and physical gift cards were distributed.

Hosting GEORGIA CEAL surveys on the Qualtrics platform proved helpful in enabling the inclusion of reCAPTCHA verification and hidden questions detectable only to bots; yet, fraudulent web-based survey responses are not solely the result of bot submissions [23]. Indeed, prior research using social media to investigate patient perceptions of patient-provider communication in a health care setting reported being similarly inundated with a large volume of low-quality and fraudulent data, noting that all study respondents passed reCAPTCHA verification, and only few (16.2%) responded to 1 or more hidden survey questions [23]. To that end, the value of web-based platforms (eg, Qualtrics and REDCap) in providing metadata, such as survey start and end times, IP addresses, and geolocation data, in addition to reCAPTCHA software and hidden question technology is clear. We further recommend the simultaneous use of multiple measures, given that isolated indicators are not equally useful at determining fraud [12,29,30]. Timestamps allow for the determination of the time taken to complete a survey and enable the exclusion of submissions with implausible response times. It is thus vital that researchers first test the survey to gauge the expected time needed for questionnaire completion and set limits for unrealistic survey response times. Still, bot sophistication may enable manipulation of timestamp information [10]. Geolocation data and IP addresses, the former often being derived from the latter [10], may be used to identify duplicate survey responses or verify geographic eligibility [2]. Yet, IP addresses and geolocations may not be ideal indicators of fraud when used alone, as some bot programs are able to bypass ballot stuffing protections and alter location data [10]. Further, surveys collected at in-person events or from persons working in the same office or building may share the same IP address; alternatively, opt-out services, web-based private networks, and other privacy services allow persons to mask their IP address and location [12,23]. In the case of the GEORGIA CEAL surveys, for example, while survey eligibility was limited to Georgian people, the proliferation of remote working arrangements during the pandemic created situations where eligible persons could have completed surveys while out of state or elsewhere.

Finally, the collection of identifiable data (eg, first and last name and email address) allows for the examination of duplicate responses (eg, multiple submissions from an individual with the same name) and suspicious submissions (eg, email addresses consisting of a random string of letters or numbers) [2]. As technological features continually evolve, however, challenges with validating email addresses must be considered. With GEORGIA CEAL Surveys I and II, for example, web-based distribution of e-gift cards necessitated that respondents provide a valid email address upon completion of the survey. However, the new *hide my email* features for Apple devices [31] and Google accounts [32] challenged email verification in many cases. Further, given the relative ease of creating new, free email accounts, 1 individual can create multiple identities and access a survey more than once [33].

#### Postdata Collection

Participant remuneration is another important consideration, and the literature is replete with suggestions for handling compensation decisions, from eliminating any participant incentive [23] to using raffle draws or lottery-style rewards [2,11,22,23], and offering “targeted appeal” incentives that are most compelling to those within the priority population (eg, classroom supplies for teachers) [1]. Prior to making study incentive-related decisions, however, knowledge of the study context and relevant state laws is important; regulations regarding raffle-style incentives in Georgia, for example, require that all persons, including those who do not consent to participate in research, be able to participate in the raffle [34]. Electronic or physical distribution of incentives requires an individual’s contact information (eg, email address and physical address) and, while such requests may limit participant anonymity, they also provide opportunities for survey authentication [10,12,22,30]. Where physical address data are collected, free applications (eg, Google Maps) or paid subscriptions (eg, Whitepages) may further assist with participant address and identity verification. Community partners have advised that studies working with vulnerable populations need to be mindful of how residential address requests may be perceived. An alternative consideration may be requesting cell phone numbers since, even though some may be less open to sharing cell phone information due to concerns about spam messages and persistent contact efforts, services like Google Voice permit anonymity while encouraging participation. We found that surveys from persons who chose not to provide an email or physical address for gift card receipts were more likely to be fraudulent. In these instances, for example, multiple submissions missing identifiers also had identical timestamps (ie, the start time and end time). These batched responses have been similarly reported by other researchers as an indicator of fraud [35].

Our experiences with GEORGIA CEAL web-based survey administration further suggest that duplicitous participants are more likely to reach out to study teams directly using abrupt language to demand remuneration; notably, however, subsequent attempts to respond and confirm participant identity often go unanswered or elicit more inadequate or unreasonable responses. Depending on available resources, the study team may also opt to dedicate 1 or 2 members to verifying respondent eligibility via telephone; however, this process is labor-intensive and associated with an increased burden for legitimate participants [23].

### Strengths and Limitations

Best practices and lessons learned following the administration of GEORGIA CEAL Survey I were incorporated into GEORGIA CEAL Survey II to facilitate a smoother and more robust survey authentication process. Yet, while adaptative monitoring (eg, openness to protocol revisions and IRB amendments) remains a strength of both surveys, fraud detection measures are numerous, and not all authentication tools described in this paper were implemented in either survey wave. Indeed, the strengths and limitations of the study design strategies and antideception tools discussed thus far underscore the utility of using *multiple* strategies throughout all research phases (ie, during survey design, data collection, data cleaning, and data analysis) to support multiple checks and flags for potentially suspicious submissions. These checks are particularly relevant during data cleaning and analyses and may be supplemented by team discussions (research staff and community partners) toward developing consensus on a protocol for removing suspicious submissions. This may involve outlining specific criteria for classifying responses as either legitimate, fraudulent, suspicious, or unclear. The creation of such criteria and protocols is important for screening submitted responses, developing exclusion decision rules, and ensuring data integrity [2]. Additionally, rigorous cleaning of data, together with the development of thorough data management protocols and data dictionaries, supports consistency, replication, data quality, and study rigor. Sensitivity analyses exploring study outcomes among subsets of data (eg, only legitimate responses vs legitimate, suspicious, and fraudulent responses) may further help gauge the implications of fraud in web-based survey research.

### Conclusions

Previous literature describes preventive versus exclusionary measures to detect and manage web-based survey fraud and calls for greater discussions about fraud management experiences and best practices in mitigating suspicious and fraudulent web-based survey participation; yet, little is known or understood about factors associated with fraudulent behavior in web-based survey research [1,10]. Further, despite ongoing bot sophistication and a dynamic technological landscape that provide new opportunities for research fraud, evidence suggests that most fraudulent data cannot be attributed to bots alone [23]. Accordingly, while scientific discussion and transparency are central to raising awareness within the research community about the magnitude of web-based data integrity threats and the availability of effective security measures, equally imperative is the need to engage community stakeholders, via approaches grounded in community-based participatory research [36], around the potential of research to influence scientific advances in order to accelerate investment in quality, legitimate survey data.

INpowerment science involves “helping stakeholders connect to the power that they already have individually and collectively and making that power more active instead of trying to bring them power” by providing “the space, resources, and access to use their power for change” [37]. GEORGIA CEAL was established in response to the COVID-19 pandemic and the need for rapid, yet, valid, community-informed, and community-owned research to guide targeted responses to a dynamic, public health crisis. As the COVID-19 landscape continues to evolve, GEORGIA CEAL has maintained its commitment to community-centered outreach, in part by capitalizing on web-based survey experiences to inform community-centered survey administration and advocate for greater community engagement, not merely as participants but coleaders in research. The GEORGIA CEAL team works closely with the GEORGIA CEAL CCB in research planning, survey creation and distribution, data analysis, and dissemination of findings to lay and scientific audiences. By thorough and practical discussion of experiences, lessons learned, and future directions for web-based survey research, we aim to *INpower* academia and communities in the defense of web-based survey data.

## Supplementary material

10.2196/51786 Multimedia Appendix 1 Garnering Effective Outreach and Research in Georgia for Impact Alliance–Community Engagement Alliance Survey II recruitment flyer.

10.2196/51786 Multimedia Appendix 2 Garnering Effective Outreach and Research in Georgia for Impact Alliance–Community Engagement Alliance Survey I questionnaire and consent form.

10.2196/51786 Multimedia Appendix 3 Garnering Effective Outreach and Research in Georgia for Impact Alliance–Community Engagement Alliance Survey II questionnaire and consent form.

10.2196/51786 Checklist 1 CHERRIES (Checklist for Reporting Results of Internet E-Surveys).

## Abbreviations

CAPTCHA: Completely Automated Public Turing Test to Tell Computers and Humans Apart
CCB: Community Coalition Board
CHERRIES: Checklist for Reporting Results of Internet E-Surveys
GEORGIA CEAL: Garnering Effective Outreach and Research in Georgia for Impact Alliance–Community Engagement Alliance
IRB: institutional review board
REDCap: Research Electronic Data Capture
