# Supplementary material for: Challenges and Lessons Learned in Managing Web-Based Survey Fraud for the Garnering Effective Outreach and Research in Georgia for Impact Alliance–Community Engagement Alliance Survey Administrations
Source: JMIR Public Health Surveill. 2024 Dec 24;10:e51786. doi: 10.2196/51786 (PMC11687484; doi:10.2196/51786)
Supplement: Multimedia Appendix 1 [file publichealth-v10-e51786-s001.pdf]

GEORGIA CEAL
Community Engagement Alliance Against COVID-19 Disparities

GA CEAL Survey II

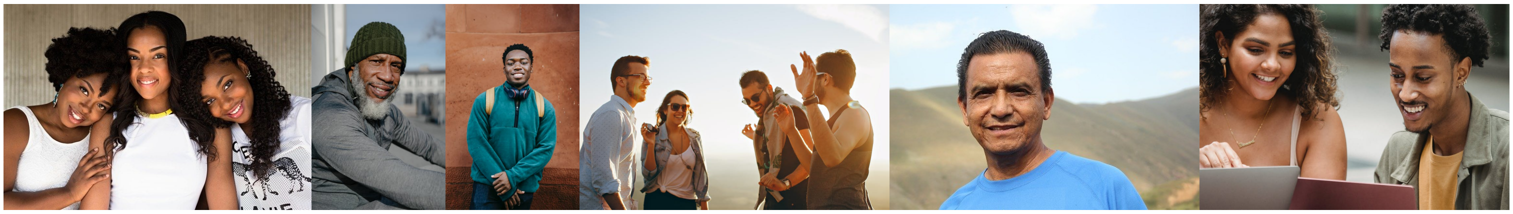

The purpose of the survey is to learn community thoughts, behaviors, and understanding regarding COVID-19, vaccines, and vaccine trials.

Eligibility Criteria

18 years of age or older

In order to participate in the survey, you must be at least 18 years of age.

Georgia residents

All survey participants must be a resident of the following counties in Georgia: Appling, Atkinson, Bacon, Baker, Brantley, Calhoun, Chattahoochee, Clarke, Clinch, Cobb, DeKalb, Dooly, Dougherty, Elbert, Fulton, Gwinnett, Hart, Henry, Jenkins, Lee, Lowndes, Oglethorpe, Randolph, Richmond, Stewart, Talbot, Telfair, Terrell, Thomas, Toombs, Twiggs, Upson, Warren, or Walton

Priority Populations

Participants must be African American or Hispanic/Latino(a).

Incentives for participants

Participants will receive a $25 e-gift card for completing the survey.

Scan the QR code below to take GA CEAL Survey II.
Please share with your friends and family.

To learn more about Georgia CEAL, contact us at georgiaceal@msm.edu
