# Supplementary material for: Challenges and Lessons Learned in Managing Web-Based Survey Fraud for the Garnering Effective Outreach and Research in Georgia for Impact Alliance–Community Engagement Alliance Survey Administrations
Source: JMIR Public Health Surveill. 2024 Dec 24;10:e51786. doi: 10.2196/51786 (PMC11687484; doi:10.2196/51786)
Supplement: Multimedia Appendix 2 [file publichealth-v10-e51786-s002.pdf]

GEORGIA Community Engagement Alliance (CEAL) Against COVID-19 Survey

Start of Block: Default Question Block

Q1 Descriptive Text
GEORGIA COMMUNITY ENGAGEMENT ALLIANCE (CEAL) AGAINST COVID-19 SURVEY SCREENING PAGE Thank you for your interest in the GEORGIA Community Engagement Alliance (CEAL) Against COVID-19 Survey. Please complete the question below to determine if you are eligible to participate.

Q2 **What county do you live in?**

- ◯ Atkinson (6)
- ◯ Baker (7)
- ◯ Calhoun (8)
- ◯ Clarke (9)
- ◯ Cobb (10)
- ◯ DeKalb (1)
- ◯ Dooly (11)
- ◯ Dougherty (12)
- ◯ Elbert (13)
- ◯ Fulton (3)
- ◯ Gwinnett (4)
- ◯ Hart (14)
- ◯ Henry (2)
- ◯ Lee (15)
- ◯ Lowndes (16)
- ◯ Oglethorpe (17)
- ◯ Terrell (18)
- ◯ Thomas (19)
- ◯ Walton (20)
- ◯ Other (5) ____

Skip To: Q3 If What county do you live in? = Atkinson

Skip To: Q3 If What county do you live in? = Baker
Skip To: Q3 If What county do you live in? = Calhoun
Skip To: Q3 If What county do you live in? = Clarke
Skip To: Q3 If What county do you live in? = Cobb
Skip To: Q3 If What county do you live in? = DeKalb
Skip To: Q3 If What county do you live in? = Dooly
Skip To: Q3 If What county do you live in? = Dougherty
Skip To: Q3 If What county do you live in? = Elbert
Skip To: Q3 If What county do you live in? = Fulton
Skip To: Q3 If What county do you live in? = Gwinnett
Skip To: Q3 If What county do you live in? = Hart
Skip To: Q3 If What county do you live in? = Henry
Skip To: Q3 If What county do you live in? = Lee
Skip To: Q3 If What county do you live in? = Lowndes
Skip To: Q3 If What county do you live in? = Oglethorpe
Skip To: Q3 If What county do you live in? = Terrell
Skip To: Q3 If What county do you live in? = Thomas
Skip To: Q3 If What county do you live in? = Walton
Skip To: Q7 If What county do you live in? = Other
Skip To: Q7 If Condition: Other Is Not Empty. Skip To: Thank you for you interest in partici....

Q3 **Are you Hispanic or Latina/o?**

- ○ No (1)
- ○ Yes (2)

Skip To: Q4 If Are you Hispanic or Latina/o? = No
Skip To: Q80 If Are you Hispanic or Latina/o? = Yes

Q80 Which of the following best describes your race? **(Please select all that apply)**

- ◯ White (11)
- ◯ Black or African American (12)
- ◯ American Indian or Alaska Native (13)
- ◯ Asian (14)
- ◯ Native Hawaiian or Pacific Islander (15)
- ◯ Other (16) ____

Skip To: Q5 If Which of the following best describes your race? (Please select all that apply) = White

Skip To: Q5 If Which of the following best describes your race? (Please select all that apply) = Black or African American

Skip To: Q5 If Which of the following best describes your race? (Please select all that apply) = American Indian or Alaska Native

Skip To: Q5 If Which of the following best describes your race? (Please select all that apply) = Asian

Skip To: Q5 If Which of the following best describes your race? (Please select all that apply) = Native Hawaiian or Pacific Islander

Skip To: Q5 If Which of the following best describes your race? (Please select all that apply) = Other

Skip To: Q5 If Condition: Other Is Not Empty. Skip To: What month were you born? .

Q4 **Which of the following best describes your race?**

- ◯ White (1)
- ◯ Black or African American (2)
- ◯ American Indian or Alaska Native (3)
- ◯ Asian (4)
- ◯ Native Hawaiian or Pacific Islander (5)
- ◯ Other (7) ____

Skip To: Q7 If Which of the following best describes your race? = White

Skip To: Q5 If Which of the following best describes your race? = Black or African American

Skip To: Q7 If Which of the following best describes your race? = American Indian or Alaska Native
Skip To: Q7 If Which of the following best describes your race? = Asian
Skip To: Q7 If Which of the following best describes your race? = Native Hawaiian or Pacific Islander
Skip To: Q7 If Which of the following best describes your race? = Other
Skip To: Q7 If Condition: Other Is Not Empty. Skip To: Thank you for your interest in partic....

Q5 What month were you born?

- January (1)
- February (2)
- March (3)
- April (4)
- May (5)
- June (6)
- July (7)
- August (8)
- September (9)
- October (10)
- November (11)
- December (12)

Skip To: Q82 If What month were you born? = January
Skip To: Q82 If What month were you born? = February
Skip To: Q82 If What month were you born? = March
Skip To: Q82 If What month were you born? = April

*

Q6 What year were you born? (Please enter as YYYY)

____

Skip To: Q7 If Condition: What year were you born? (P... Is Greater Than 2003. Skip To: Thank you for your interest in partic....

Skip To: Q8 If Condition: What year were you born? (P... Is Less Than 2003. Skip To: GEORGIA COMMUNITY ENGAGEMENT ALLIANCE....

Skip To: Q7 If Condition: What year were you born? (P... Is Equal to 2003. Skip To: Thank you for your interest in partic....

Q82 What year were you born? (Please enter as YYYY)

____

Skip To: Q7 If Condition: What year were you born? (P... Is Greater Than 2003. Skip To: Thank you for your interest in partic....

Skip To: Q8 If Condition: What year were you born? (P... Is Less Than or Equal to 2003. Skip To: GEORGIA COMMUNITY ENGAGEMENT ALLIANCE....

Page Break

Q7 Descriptive Text
Thank you for your interest in participating in the GEORGIA COMMUNITY ENGAGEMENT ALLIANCE (CEAL) Against COVID-19 Survey. Unfortunately, you do not qualify to participate in this survey. For more information, please contact GEORGIA CEAL at georgiaceal@msm.edu and/or call 404-752-1144. Thank you for your time. Have a great day!

Skip To: End of Survey If Thank you for your interest in participating in the GEORGIA COMMUNITY ENGAGEMENT ALLIANCE (CEAL)... Is Displayed

Q8 **GEORGIA COMMUNITY ENGAGEMENT ALLIANCE (CEAL) AGAINST COVID-19 SURVEY**

Thank you for your interest in the GEORGIA Community Engagement Alliance (CEAL) Against COVID-19 Community. We are inviting you to participate in this survey because you: 1) identify as Black or African American or Latinx; 2) live in priority counties and are 3) at least 18 years old. The purpose of the survey is to learn community thoughts, behaviors, and understanding regarding COVID-19, vaccines, and vaccine trials. It is part of a larger research study for community-engaged outreach and response to COVID-19 called GEORGIA (Garnering Effective Outreach and Research in Georgia for Impact Alliance) CEAL. We are inviting 2000 eligible African American/Blacks or Latinx people in Georgia to answer this survey.

If you decide to take part in the study, we will ask you to complete a 15-20 minute-long survey. You are asked to answer each question. We ask you to give your name, email, or address to get a $25 gift card as a thank you for your time. You do not have to provide us with your name, email, or address to join, but we will not be able to send the gift card without it. Risks/Benefits: There are no known risks from being in this study, and you will not benefit personally. However, we hope that others may benefit in the future from what we learn because of this study. You will not have any costs associated with participating in this survey. We will keep the information you provide confidential; however federal regulatory agencies and the Morehouse School of Medicine Institutional Review Board (a committee that reviews and approves research studies) may inspect and copy records pertaining to this research. If we write a report about this study, we will do so in such a way that you cannot be identified. Once the survey is closed, we will destroy any data that can identify you. We cannot promise total privacy of data about you. The Morehouse School of Medicine's Institutional Review Board -- the committee that approved this project -- may have access to these records. We will not identify you in any way as being in this research in any papers in public health or other journals. We will not pinpoint you in any reports made on this research at public meetings. Your answers will not be shared with anyone or stored in a way that they can be traced back to you. Your responses may be part of a research report, but your name and any other data that could give away who you are will not be used. We will only use your contact information to send you the electronic gift card. If you have any questions about the research study itself, please contact us at GEORGIACEAL@msm.edu or call 404-752-1700.

Do you consent (agree) to take this survey? Please select one option to continue. If you answer NO, you are not eligible to take this survey. Thank you for your time.

- ◯ YES, I consent (agree) to take this survey. (4)
- ◯ NO, I do not consent (agree) to take this survey. (5)

Skip To: End of Survey If GEORGIA COMMUNITY ENGAGEMENT ALLIANCE (CEAL) AGAINST COVID-19 SURVEY Thank you for your interes... = NO, I do not consent (agree) to take this survey.

Q9 Descriptive Text
Knowledge and Misinformation about COVID-19

Q10 Please indicate whether you believe these statements to be true or false. If you do not know the answer, please select "Don't Know."

	True (1)	False (2)	Don't Know (3)
a. COVID-19 is spread through coughing and sneezing. (1)			
b. Wearing a cloth face covering may prevent you from spreading COVID-19 to someone else. (2)			
c. Regularly washing your hands for 20 seconds can help protect you from COVID-19. (3)			
d. People exposed to COVID-19 can spread the disease to others, even if they do not have any symptoms. (4)			
e. Currently, there is a vaccine available to prevent COVID-19 infection that has been approved by the US Food and Drug Administration. (5)			
f. Currently, there is a cure for COVID-19. (6)			
g. Children cannot transmit COVID-19 to other people. (7)			
h. Wearing a face mask is harmful to your health. (8)			
i. Hydroxychloroquine is an effective treatment for COVID-19. (9)			

j. Anyone can get sick with COVID-19. (10)	○	○	○
k. In the U.S., COVID-19 is affecting all race and ethnicity groups equally. (11)	○	○	○
l. In the U.S., COVID-19 has affected Black, Hispanic/Latino, and Native American populations at a higher rate than White populations. (12)	○	○	○
m. Getting a vaccine for a disease means you might get sick from the vaccine itself. (13)	○	○	○
n. A vaccine is made of small amounts of the disease and therefore can make you sick. (14)	○	○	○
o. Getting vaccinated for a disease helps to protect you from getting it. (15)	○	○	○
p. When you get vaccinated for a disease, it helps to protect others from getting the disease. (16)	○	○	○
q. Getting a vaccine helps your body attack that disease if exposed to that disease in the future. (17)	○	○	○
r. Getting a vaccine protects you and means you cannot get the disease if exposed to it in the future. (18)	○	○	○

Q11 Descriptive Text
Trusted Sources of Information about COVID-19

Q12 How much do you trust each of these sources to provide correct information about COVID-19? **(Select one response for each row.)**

	Not at all (1)	A little (2)	A great deal (3)	Don't Know (4)
Your doctor or health care provider (1)	○	○	○	○
Your faith leader (e.g., pastor, priest, etc.) (2)	○	○	○	○
Your close friends and members of your family (3)	○	○	○	○
People you go to work or class with or other people you know (4)	○	○	○	○
News on the radio, TV, online, or in newspapers (5)	○	○	○	○
Your contacts on social media (6)	○	○	○	○
The U.S. government (7)	○	○	○	○
The U.S. Coronavirus Task Force (8)	○	○	○	○

Q13 Descriptive Text
COVID-19 Information Seeking

Q14 In the last week have you looked for any information about COVID-19?

○ Yes (1)

○ No (2)

Skip To: Q16 If In the last week have you looked for any information about COVID-19? = No

Q15 In the last week, which of the following sources have you used to get information about COVID-19? **(Select all that apply)**

- ▢ Twitter (1)
- ▢ Facebook (2)
- ▢ Newspaper (3)
- ▢ Friends or family members (4)
- ▢ Coworkers or classmates (5)
- ▢ Doctor of other heath care providers/professionals (6)
- ▢ Official government websites (7)
- ▢ The President (8)
- ▢ State Governor (9)
- ▢ Mayor (10)
- ▢ World Health Organization (WHO) (11)
- ▢ Centers for Disease Control and Prevention (CDC) (12)
- ▢ National Institutes of Health (NIH) (13)
- ▢ State, County, or City Health Department (14)
- ▢ CNN (15)
- ▢ Fox News (16)
- ▢ MSNBC (17)

- ☐ Local news station (e.g., CBS, ABC, NBC) (18)
- ☐ NPR (19)
- ☐ Complementary and alternative health websites (20)
- ☐ Complementary and alternative health providers (e.g., chiropractors, naturopaths, etc.) (21)
- ☐ Faith-based Institutions (22)
- ☐ Mainstream health websites (e.g., Web MD) (23)
- ☐ Local Universities (e.g., Morehouse School of Medicine, Emory, etc.) (24)

Q16 Descriptive Text
COVID-19 Prevention Coronavirus Disease 2019 (COVID-19) is a disease caused by the new coronavirus. The first set of questions asks what you think about COVID-19 and how to stay safe from COVID-19.

Q17 In the past 7 days, how often have you chosen to do each of the following when in public to keep yourself and others safe from COVID-19? *(Do not include things you were required to do, such as wear a mask while visiting a store. Select one response for each row.)*

	All the time (1)	Very Often (2)	Some of the time (3)	Never (4)
Wore a face covering or mask (1)	○	○	○	○
Washed my hands with soap or used hand sanitizer several times per day (2)	○	○	○	○
Stayed at least 6 feet away from other people who are not from my household (3)	○	○	○	○

Q18 Descriptive Text
COVID-19 Testing

Q19 Have you ever been tested for COVID-19?

- ◯ Yes (1)
- ◯ No (2)

Skip To: Q22 If Have you ever been tested for COVID-19? = No

Q20 How easy or hard was it to get the COVID-19 test?

- ◯ Very easy (1)
- ◯ Easy (2)
- ◯ Hard (3)
- ◯ Very Hard (4)

Q21 Have you ever had a positive COVID-19 test positive result?

- ◯ I had COVID-19 (1)
- ◯ I did NOT have COVID-19 (2)
- ◯ Do not have results yet (3)
- ◯ Prefer not to answer (4)

Skip To: Q23 If Have you ever had a positive COVID-19 test positive result? = I had COVID-19
Skip To: Q23 If Have you ever had a positive COVID-19 test positive result? = I did NOT have COVID-19
Skip To: Q23 If Have you ever had a positive COVID-19 test positive result? = Do not have results yet
Skip To: Q23 If Have you ever had a positive COVID-19 test positive result? = Prefer not to answer

---

Q22 Why have you not been tested for COVID-19? **(Select all that apply).**

- ☐ I haven't felt sick. (1)
- ☐ I felt sick, but I didn't feel sick enough to get tested. (2)
- ☐ I felt sick, but my health care provider told me to just stay at home and away from other people. (3)
- ☐ I was told that testing wasn't available. (4)
- ☐ I didn't have a way to get to the testing location. (5)
- ☐ I didn't have the money to pay for a test. (6)
- ☐ I didn't know where to go to get tested. (7)
- ☐ I didn't have someone to watch my children or other people in my care so I could go get tested. (8)
- ☐ I couldn't take time off work to get tested. (9)
- ☐ I'm afraid that a positive test result will mean that I have to miss work. (10)
- ☐ I'm afraid to get a COVID-19 test. (11)
- ☐ I don't trust researchers. (12)
- ☐ I don't trust the government. (13)
- ☐ Other reason: (14) ____

Q23 Descriptive Text
Perceived Risk and Severity of COVID-19

Q24 How would you rate your own risk of getting COVID-19 – do you think there’s a high risk of that, a moderate risk, a low risk or no risk?

- ○ High risk (1)
- ○ Moderate risk (2)
- ○ Low risk (3)
- ○ No risk (4)
- ○ Don't Know/No opinion (5)
- ○ Not Applicable/Refused (6)
- ○ I have previously tested positive for COVID-19 (7)

Q25- Descriptive Text
Intention to Get COVID-19 Vaccine The next question asks about a COVID-19 vaccine. A vaccine is a substance that helps protect against certain diseases.

Q26 Have you received a COVID-19 Vaccine?

- ○ No (1)
- ○ Yes, 1 of 1 required dose (2)
- ○ Yes, 1 of 2 required doses (3)
- ○ Yes, 2 of 2 required doses (4)

Skip To: Q27 If Have you received a COVID-19 Vaccine? = No
Skip To: Q28 If Have you received a COVID-19 Vaccine? = Yes, 1 of 1 required dose
Skip To: Q28 If Have you received a COVID-19 Vaccine? = Yes, 1 of 2 required doses
Skip To: Q28 If Have you received a COVID-19 Vaccine? = Yes, 2 of 2 required doses

Q27

How likely are you to get an approved COVID-19 vaccine when it becomes available?

Not at all likely					Neutral					Extremely likely
0	1	2	3	4	5	6	7	8	9	10

Q28 Descriptive Text

Reasons for Getting/Not Getting a COVID-19 Vaccine

Q29 Why would/did you get a COVID-19 vaccine? **(Select all that apply.)**

- ▢ I want to keep my family safe. (1)
- ▢ I want to keep my community safe. (2)
- ▢ I want to keep myself safe. (3)
- ▢ I have a chronic health problem, like asthma or diabetes. (4)
- ▢ My doctor told me to get a COVID-19 vaccine. (5)
- ▢ I don't want to get really sick from COVID-19. (6)
- ▢ I want to feel safe around other people. (7)
- ▢ I believe life won't go back to normal until most people get a COVID-19 vaccine. (8)
- ▢ I do NOT plan on getting a COVID-19 vaccine (9)
- ▢ Other: (10) ____

Skip To: Q31 If Why would/did you get a COVID-19 vaccine? (Select all that apply.) = I want to keep my family safe.

Skip To: Q31 If Why would/did you get a COVID-19 vaccine? (Select all that apply.) = I want to keep my community safe.

Skip To: Q31 If Why would/did you get a COVID-19 vaccine? (Select all that apply.) = I want to keep myself safe.

Skip To: Q31 If Why would/did you get a COVID-19 vaccine? (Select all that apply.) = I have a chronic health problem, like asthma or diabetes.

Skip To: Q31 If Why would/did you get a COVID-19 vaccine? (Select all that apply.) = My doctor told me to get a COVID-19 vaccine.

Skip To: Q31 If Why would/did you get a COVID-19 vaccine? (Select all that apply.) = I don't want to get really sick from COVID-19.

Skip To: Q31 If Why would/did you get a COVID-19 vaccine? (Select all that apply.) = I want to feel safe around other people.

Skip To: Q31 If Why would/did you get a COVID-19 vaccine? (Select all that apply.) = I believe life won't go back to normal until most people get a COVID-19 vaccine.

Skip To: Q30 If Why would/did you get a COVID-19 vaccine? (Select all that apply.) = I do NOT plan on getting a COVID-19 vaccine

Skip To: Q31 If Condition: Other: Is Not Empty. Skip To: COVID-19 Clinical

Q30 Why would you NOT get a COVID-19 vaccine? **(Select all that apply.)**

- ☐ I'm allergic to vaccines. (1)
- ☐ I don't like needles. (2)
- ☐ I'm not concerned about getting really sick from COVID-19. (3)
- ☐ I'm concerned about the side effects from the vaccine. (4)
- ☐ I don't think vaccines work very well. (5)
- ☐ I don't trust that the vaccine will be safe. (6)
- ☐ I don't believe the COVID-19 pandemic is as bad as some people say it is. (7)
- ☐ I don't want to pay for it. (8)
- ☐ I don't know enough about how well a COVID-19 vaccine works. (9)
- ☐ I don't trust researchers. (10)
- ☐ I don't trust the government. (11)
- ☐ The COVID 19 vaccine may not be safe. (12)
- ☐ It's too hard to get. (13)
- ☐ It will take too much time. (14)
- ☐ Vaccines, in general, are bad for you. (15)
- ☐ I want my body to build its own immunity. (17)
- ☐ Other: (16) ____

Q31- Descriptive Text

COVID-19 Clinical Trials Now we are going to ask you some questions about COVID-19 clinical trials. A **clinical trial** is a kind of research study that tests whether medical products like medicines, vaccines, or devices are safe and effective in people. Clinical trials might test a new vaccine to see if it works and if it is safe, or compare a new treatment with an existing one. These studies may show which medical products and approaches work best for certain illnesses or groups of people. Right now, clinical trials are being done across the U.S. to see if new treatments and vaccines for COVID-19 work to keep people healthy.

Q32 Have you ever signed up for a COVID-19 clinical trial?

- ☐ Yes, I signed up for a clinical trial for a COVID-19 vaccine (1)
- ☐ Yes, I signed up for a clinical trial for a COVID-19 treatment (2)
- ☐ No, I have never signed up for a COVID-19 clinical trial (3)

Skip To: Q33 If Have you ever signed up for a COVID-19 clinical trial? = No, I have never signed up for a COVID-19 clinical trial

Skip To: Q40 If Have you ever signed up for a COVID-19 clinical trial? = Yes, I signed up for a clinical trial for a COVID-19 vaccine

Skip To: Q40 If Have you ever signed up for a COVID-19 clinical trial? = Yes, I signed up for a clinical trial for a COVID-19 treatment

Q33 Descriptive Text

Awareness and Knowledge about COVID-19 Clinical Trials

Q34 Are you aware of COVID-19 clinical trials that are being done?

- [ ] Yes, clinical trials for COVID-19 vaccines (1)
- [ ] Yes, clinical trials for COVID-19 treatments (2)
- [ ] No (3)
- [ ] Not Sure (4)

Q35 Do you know what to do to sign up for a COVID-19 clinical trial in your area?

- ○ Yes (1)
- ○ No (2)
- ○ Not sure (3)

Q36 Descriptive Text
Willingness and Intentions to Register or Enroll in a COVID-19 Clinical Trial We have a few more questions about COVID-19 clinical trials. Again, a **clinical trial** is a kind of research study. Clinical trials study if treatments or vaccines are safe for people and if they work like they are supposed to. Right now, clinical trials are being done across the U.S. to see if new treatments and vaccines for COVID-19 work to keep people healthy.

Q37
How **willing** (eager) are you to sign up for a clinical trial for a COVID-19 vaccine?

Not at all willing					Neutral					Extremely likely
0	1	2	3	4	5	6	7	8	9	10

Q38

How **likely** (what are the chances) are you to sign up for a clinical trial for a COVID-19 vaccine?

Not at all likely					Neutral					Extremely likely
0	1	2	3	4	5	6	7	8	9	10

Q39 Why would you NOT take part in a clinical trial for a COVID-19 vaccine? **(Select all that apply.)**

- I don’t trust researchers. (1)
- I don’t trust the government. (2)
- The COVID-19 vaccine may not be safe. (3)
- I don’t have a way to get to the trial (4)
- I don’t believe clinical trials are important. (5)
- I don’t understand what will happen to me. (6)
- It will cost me money. (7)
- It will cost me time. (8)
- Vaccines in general are bad for you. (9)
- I have health problems that prevent me from taking part in a clinical trial. (10)
- Other reason: (11) ____

Q40- Descriptive Text

Trust Regarding COVID-19 Clinical Trials

Q41 Below are sources of information of COVID-19 clinical trials. How much do you trust each of these sources to give correct information? **(Select one response for each row).**

	A great deal (1)	A fair amount (2)	Not very much (3)	None at all (4)	No opinion (5)
The U.S. government (1)	○	○	○	○	○
Your doctor or health care provider (2)	○	○	○	○	○
Your local health care clinic or hospital (3)	○	○	○	○	○
University hospitals (4)	○	○	○	○	○
Companies that make drugs for medical use (5)	○	○	○	○	○
People who do research (6)	○	○	○	○	○

Q42 Descriptive Text
Underrepresented Group Participation in Clinical Trials

Q43 Please indicate your level of agreement with the following statements.

	Strongly Disagree (1)	Disagree (2)	Neutral (3)	Agree (4)	Strongly Agree (5)
a. Participants in any clinical trial should represent all the people who may use the medicine, vaccine, or device being tested. (1)	○	○	○	○	○
b. To make sure that COVID-19 vaccines are safe and effective for all people regardless of race/ethnicity, people from different racial/ethnic backgrounds must participate in the clinical trials that are testing these vaccines (2)	○	○	○	○	○

Q44 Descriptive Text

Trust in Medical Researchers

Q45 Please indicate your level of agreement with the following statements.

	Strongly Disagree (1)	Disagree (2)	Neutral (3)	Agree (4)	Strongly Agree (5)
a. To get people to take part in a study, medical researchers usually do not explain all the dangers about participation. (1)	○	○	○	○	○
b. Participants should be concerned about being deceived or misled by medical researchers. (2)	○	○	○	○	○
c. Medical researchers act differently toward minority subjects than toward white subjects. (3)	○	○	○	○	○
d. Medical researchers unfairly select minorities for their most dangerous research studies. (4)	○	○	○	○	○
e. Medical researchers are generally honest in telling participants about different treatment options available for their conditions. (5)	○	○	○	○	○

f. Medical researchers would not conduct experiments on people without their knowledge today. (6)	○	○	○	○	○
g. Most medical researchers would not lie to people to try to convince them to participate in a research study. (7)	○	○	○	○	○
h. In general, medical researchers care more about doing their research than about the participants' medical needs. (8)	○	○	○	○	○

Page Break

Q46 Descriptive Text
The next set of questions asks about you and your household.
Access to Health Services

Q47 When was the last time you saw a doctor or other health care professional for a physical or regular check-up? Do not include visits when you were sick.

- ○ Never (1)
- ○ Within the past 12 months/ 1 year (2)
- ○ 1 to 2 years ago (3)
- ○ 3 to 4 years ago (4)
- ○ 5 to 9 years ago (5)
- ○ 10 years ago (6)

Q48 Is there a place that you usually go when you are sick?

- ○ Yes (1)
- ○ No (2)
- ○ Don't Know (3)

Skip To: Q49 If Is there a place that you usually go when you are sick? = Yes
Skip To: Q50 If Is there a place that you usually go when you are sick? = No
Skip To: Q50 If Is there a place that you usually go when you are sick? = Don't Know

---

Q49 What kind of place do you go most often for medical care?

- Community Clinic or health center (1)
- Family doctor (2)
- Hospital ER (3)
- Urgent care clinic at a hospital (4)
- Urgent care clinic not at a hospital (5)
- Retail Center (for example, in a drug store) (6)
- Some other place (7)
- There is no one place I go to most often for medical care (8)

Q50 Do you have any kind of health insurance or health care plan?

- Yes (1)
- No (2)
- Don't know (3)

Skip To: Q51 If Do you have any kind of health insurance or health care plan? = No

Skip To: Q53 If Do you have any kind of health insurance or health care plan? = Don't know

Skip To: Q52 If Do you have any kind of health insurance or health care plan? = Yes

---

Q51 Did you lose health care coverage because of the COVID-19 pandemic?

- Yes (1)
- No (2)

Skip To: Q53 If Did you lose health care coverage because of the COVID-19 pandemic? , Yes Is Displayed

---

Q52 What is the primary kind of health insurance or health care plan that you have now?

- ○ Private health insurance through a job or school (1)
- ○ Insurance bought through a government exchange such as healthcare.gov (2)
- ○ Insurance bought from a health plan or company (3)
- ○ Medicare (4)
- ○ Medi-Gap (5)
- ○ Medicaid (6)
- ○ CHIP or kid's state insurance (7)
- ○ Military health care (8)
- ○ Indian Health Service (9)
- ○ Other (10) ____
- ○ Don't know (11)

Q53 The COVID-19 pandemic may cause challenges for some people, whether they get COVID-19 or not. In the past month have you or your family experienced any of the challenges below? **(Select one response for each row.)**

	No, this is not a challenge (1)	Yes, this is a minor challenge (2)	Yes, this is a major challenge (3)
Getting the health care, I need (including for mental health) (1)	○	○	○
Having a place to live (2)	○	○	○
Getting enough food to eat (3)	○	○	○
Having clean water to drink (4)	○	○	○
Getting the medicine, I need (5)	○	○	○
Getting to where I need to go (6)	○	○	○

Q54 Descriptive Text

Demographics

Q55 Were you born in the U.S.?

○ Yes (1)

○ No (2)

○ Prefer not to answer (3)

Q56 What is your gender?

- ○ Man (1)
- ○ Woman (2)
- ○ Transgender female or trans woman (3)
- ○ Transgender male or trans man (4)
- ○ Nonbinary, genderqueer, or genderfluid (5)
- ○ I would describe my gender as: (6) ____
- ○ Prefer not to answer (7)

Q57 Which of the following best describes how you think of yourself?

- ○ Gay (1)
- ○ Lesbian (2)
- ○ Straight (that is, not gay or lesbian) (3)
- ○ Bisexual (4)
- ○ Other (5)
- ○ Prefer not to answer (6)

Q58 Which of the following best describes your race? (**Please select all that apply).**

- ☐ White (1)
- ☐ Black or African American (3)
- ☐ Asian (4)
- ☐ American Indian or Alaska Native (5)
- ☐ Native Hawaiian or Pacific Islander (6)
- ☐ Other (8) ____
- ☐ Prefer not to answer (7)

Q59 What is the highest degree or level of school you have completed?

- ○ Less than high school (1)
- ○ Some high school (2)
- ○ High school graduate or GED (3)
- ○ Associate’s or technical degree (for example, AA or AS) (4)
- ○ Bachelor’s degree (for example BA, BS, or AB) (5)
- ○ Graduate degree (for example MA, PhD) (6)
- ○ Prefer not to answer (7)

Q60 In 2019, what was your total household income before taxes?

- Less than $15,000 (1)
- $15,000 – $19,999 (2)
- $20,000 – $24,999 (3)
- $25,000 – $34,999 (4)
- $35,000 – $49,999 (5)
- $50,000 – $74,999 (6)
- $75,000 – $99,999 (7)
- $100,000 and above (8)
- Prefer not to answer (9)

Q61 How many people live or stay in your household right now? Include yourself, any other adults, and any children.

- ○ 1 (4)
- ○ 2 (5)
- ○ 3 (6)
- ○ 4 (7)
- ○ 5 (8)
- ○ 6 (9)
- ○ 7 (10)
- ○ 8 (11)
- ○ 9 (12)
- ○ 10 (13)
- ○ 11 (14)
- ○ 12 (15)
- ○ 13 (16)
- ○ 14 (17)
- ○ 15 (18)
- ○ 16 (19)
- ○ 17 (20)
- ○ 18 (21)
- ○ 19 (22)
- ○ 20 (23)

Q62 Which of the options describes your situation right now? **(Select all that apply.)**

- ☐ Working for pay—part time (less than 40 hours a week) (1)
- ☐ Working for pay—full time (40 hours a week or more) (2)
- ☐ Working without pay (for example, as an intern) (3)
- ☐ On leave from work (4)
- ☐ Unemployed and looking for a job (5)
- ☐ Unemployed and NOT looking for a job (6)
- ☐ Retired from work (7)
- ☐ Staying at home, taking care of the home or of others (8)
- ☐ Not able to work because of a disability (9)
- ☐ Going to school (10)
- ☐ Other: (11) ____

Q63 Descriptive Text
Spoken Languages

Q64 Do you speak a language other than English at home?

- ○ Yes (1)
- ○ No (2)
- ○ Prefer not to answer (3)

Skip To: Q67 If Do you speak a language other than English at home? = Yes
Skip To: Q69 If Do you speak a language other than English at home? = No
Skip To: Q69 If Do you speak a language other than English at home? = Prefer not to answer

---

Q65 What other language(s) do you speak. **(Select ALL that apply)**

- ☐ Spanish (1)
- ☐ Chinese (incl. Cantonese, Mandarin, other Chinese languages) (2)
- ☐ French and French Creole (3)
- ☐ Tagalog (4)
- ☐ Vietnamese (5)
- ☐ Korean (6)
- ☐ German (7)
- ☐ Arabic (8)
- ☐ Russian (9)
- ☐ Italian (10)
- ☐ Portuguese (11)
- ☐ Hindi (12)
- ☐ Polish (13)
- ☐ Japanese (14)
- ☐ Urdu (15)
- ☐ Persian (16)
- ☐ Gujarati (17)
- ☐ Greek (18)

- ☐ Bengali (19)
- ☐ Panjabi (20)
- ☐ Telugu (21)
- ☐ Armenian (22)
- ☐ Hmong (23)
- ☐ Hebrew (24)
- ☐ Yoruba (25)
- ☐ Amharic/Somali (26)
- ☐ Igbo (27)
- ☐ Twi (28)
- ☐ Swahili (29)
- ☐ Other: (30) ____

Page Break

Q66 Descriptive Text
Reading Ability

Q67 How often do you need someone to help you read written information from your doctor or drug store?

- ○ Never (1)
- ○ Rarely (2)
- ○ Sometimes (3)
- ○ Often (4)
- ○ Always (5)

Q68 What is your zip code?

____

Q69 Descriptive Text
If you wish to receive a $25 gift card to thank you for your time for participating in this survey please include your name, email, and address in fields below. Please check your email twice to confirm that you have entered it correctly so that we may send the electronic gift card to the correct email address.

Q70 Name

____

Q71 Email Address

____

Q72 Mailing Address

____

Q73 Please confirm your email address by typing it again below.

____

Q74 Which electronic gift card would you like?

- ○ Amazon (1)
- ○ Walmart (2)

Q75 THANK YOU FOR YOUR INTEREST IN GEORGIA CEAL. For more information, please contact GEORGIA CEAL at georgiaceal@msm.edu and or 404-752-1144.

Please click the arrow to submit survey.

End of Block: Default Question Block
