# Supplementary material for: Challenges and Lessons Learned in Managing Web-Based Survey Fraud for the Garnering Effective Outreach and Research in Georgia for Impact Alliance–Community Engagement Alliance Survey Administrations
Source: JMIR Public Health Surveill. 2024 Dec 24;10:e51786. doi: 10.2196/51786 (PMC11687484; doi:10.2196/51786)
Supplement: Multimedia Appendix 3 [file publichealth-v10-e51786-s003.pdf]

CEAL Survey II

Start of Block: Default Question Block

Q1
CEAL Survey II- GEORGIA Community Engagement Alliance (CEAL) Against COVID-19 SCREENING PAGE
(Para llenar esta encuesta en español por favor seleccione 'Español' en la lista desplegable del cuadro que está a la derecha)

Thank you for your interest in the GEORGIA Community Engagement Alliance (CEAL) Against COVID-19 Survey. Please complete the questions on the next page to determine if you are eligible to participate.

JS

Q2 What number comes after 59?

- ○ 58 (1)
- ○ 60 (2)
- ○ 61 (3)

Q3 You are not eligible to complete this survey. Thank you

Q4 What county do you live in?

- Appling (35)
- Atkinson (2)
- Bacon (36)
- Baker (4)
- Brantley (37)
- Calhoun (6)
- Chattahoochee (38)
- Clarke (8)
- Clinch (9)
- Cobb (10)
- DeKalb (11)
- Dooly (12)
- Dougherty (13)
- Elbert (14)
- Fulton (15)
- Gwinnett (16)
- Hart (17)
- Henry (18)
- Jenkins (19)
- Lee (20)
- Lowndes (21)

- ○ Oglethorpe (22)
- ○ Randolph (23)
- ○ Richmond (24)
- ○ Stewart (25)
- ○ Talbot (26)
- ○ Telfair (27)
- ○ Terrell (28)
- ○ Thomas (29)
- ○ Toombs (30)
- ○ Twiggs (31)
- ○ Upson (32)
- ○ Warren (39)
- ○ Walton (33)
- ○ Other (34) ____

Q5 Are you Hispanic or Latina/o?

- ○ No (1)
- ○ Yes (2)

Q6 Which of the following best describes your race? (Please select all that apply)

- ▢ White (1)
- ▢ Black or African American (2)
- ▢ American Indian or Alaska Native (3)
- ▢ Asian (4)
- ▢ Native Hawaiian or Pacific Islander (5)
- ▢ Other ______ (6) ____

Q7 Which of the following best describes your race?

- ○ White (1)
- ○ Black or African American (2)
- ○ American Indian or Alaska Native (3)
- ○ Asian (4)
- ○ Native Hawaiian or Pacific Islander (5)
- ○ Other ______ (6) ____

Q8 What month were you born?

- ◯ January (1)
- ◯ February (2)
- ◯ March (3)
- ◯ April (4)
- ◯ May (5)
- ◯ June (6)
- ◯ July (7)
- ◯ August (8)
- ◯ September (9)
- ◯ October (10)
- ◯ November (11)
- ◯ December (12)

Q9 What year were you born? (Please enter as YYYY)

____

Q10 What year were you born? (Please enter as YYYY)

____

Q11 Thank you for your interest in participating in the GEORGIA COMMUNITY ENGAGEMENT ALLIANCE (CEAL) Against COVID-19 Survey II. Unfortunately, you do not qualify to participate

in this survey. For more information, please contact GEORGIA CEAL at georgiaceal@msm.edu and/or call 404-752-1700. Thank you for your time. Have a great day!

Page Break

Q12 **GEORGIA CEAL AGAINST COVID-19 Disparities SURVEY II**

Thank you for your interest in the Garnering Effective Outreach and Research in Georgia for Impact Alliance (GEORGIA) Community Engagement Alliance (CEAL) Against COVID-19 Disparities Community Survey. We are inviting you to participate in this survey because you: 1) identify as Black or African American or Latina/o; 2) live in one of our priority counties and are 3) at least 18 years old. The purpose of the survey is to learn about community thoughts, behaviors, and understanding regarding COVID-19, vaccines, and vaccine trials. This survey is part of a larger research study for community-engaged outreach and response to COVID-19 under the GEORGIA CEAL. We are inviting about 2,700 eligible African American/Blacks or Latina/o adults in Georgia to answer this survey. If you decide to take part in the study, we will ask you to complete a survey that will take about 20-minutes. We will provide a $25 gift card to participants that complete the survey. All surveys will be authenticated prior to the $25 gift card being sent out. We ask you to give your name, email, and mailing address to get your gift card as a thank you for your time. You do not have to give us your name, email, and mailing address to complete the survey, but we will not be able to send the gift card without it. Risks/Benefits: There are no known risks to being in this study and you may not benefit personally. However, we hope that others may benefit in the future from what we learn because of this study. You will not have any costs associated with participating in this survey. We cannot promise total confidentiality of data about you. The federal regulatory agencies and the Morehouse School of Medicine Institutional Review Board (a committee that reviews and approves research studies) may inspect and copy records pertaining to this research. If we write a report about this study, we will do so in such a way that you cannot be identified. We will not identify you in any way as being in this research in any papers in scientific or other journals, reports, or presentations at meetings. Your responses may be part of a research report, but your name and any other data that could give away who you are will not be used. Once the survey is closed and the data verified, we will destroy any data that can identify you. We will only use your contact information to send you the gift card and information about COVID-19. If you have any questions about the research study itself, please contact us at GEORGIACEAL@msm.edu or call 404-752-1700.

Do you consent (agree) to take this survey? Please select one option to continue. If you answer NO, you are not eligible to take this survey. Thank you for your time.

○ YES, I consent (agree) to take this survey. (1)

○ NO, I do not consent (agree) to take this survey. (2)

Q13 **Knowledge and Misinformation about COVID-19**

Please indicate whether you believe these statements to be true or false. If you do not know the answer, please select “Don’t Know.”

	True (1)	False (2)	Don't Know (3)
a. COVID-19 is spread through coughing and sneezing. (1)	o	o	o
b. Wearing a cloth face covering may prevent you from spreading COVID-19 to someone else. (2)	o	o	o
c. Regularly washing your hands for 20 seconds can help protect you from COVID-19. (3)	o	o	o
d. People exposed to COVID-19 can spread the disease to others, even if they do not have any symptoms. (4)	o	o	o
e. Currently, there is a vaccine available to prevent COVID-19 infection that has been approved by the US Food and Drug Administration. (5)	o	o	o
f. Currently, there is a cure for COVID-19. (6)	o	o	o
g. Children cannot transmit COVID-19 to other people. (7)	o	o	o
h. Wearing a face mask is harmful to	o	o	o

your health. (8)			
i. Hydroxychloroquine (Plaquenil) is an effective treatment for COVID-19. (9)	○	○	○
j. Anyone can get sick with COVID-19. (10)	○	○	○
k. In the U.S., COVID-19 is affecting all race and ethnicity groups equally. (11)	○	○	○
l. In the U.S., COVID-19 has affected Black, Hispanic/Latino, and Native American populations at a higher rate than White populations. (12)	○	○	○
m. Getting a vaccine for a disease means you might get sick from the vaccine itself. (13)	○	○	○
n. A vaccine is made of small amounts of the disease and therefore can make you sick. (14)	○	○	○
o. Getting vaccinated for a disease helps to protect you from getting it. (15)	○	○	○
p. When you get vaccinated for a disease, it helps to protect others from getting the disease. (16)	○	○	○
q. Getting a vaccine helps your body attack that disease if exposed to that	○	○	○

disease in the future. (17)			
r. Getting a vaccine protects you and means you cannot get the disease if exposed to it in the future. (18)	○	○	○
s. Ivermectin is effective to prevent or treat for COVID-19. (19)	○	○	○
t. CDS (Chlorine Dioxide; Miracle Mineral Solution-MMS) is effective to prevent or treat COVID-19. (20)	○	○	○

Q14 **Trusted Sources of Information about COVID-19**

How much do you trust each of these sources to provide correct information about COVID-19? (Select one response for each row.)

	Not at all (1)	A little (2)	A great deal (3)	Don't Know (4)	Does Not Apply (5)
Your doctor or health care provider (1)	○	○	○	○	○
Your faith leader (e.g., pastor, priest, minister, rabbi etc.) (2)	○	○	○	○	○
People you go to work or class with or other people you know (3)	○	○	○	○	○
News on the radio, TV, online, or in newspapers (4)	○	○	○	○	○
Your contacts on social media (5)	○	○	○	○	○
The federal government (6)	○	○	○	○	○
State and/or local government (7)	○	○	○	○	○
Tribal Leadership (8)	○	○	○	○	○
The Centers for Disease	○	○	○	○	○

Control and Prevention (CDC) (9)					
A community organization that provides services and assistance where you live (10)	○	○	○	○	○
Your close friends and members of your family (11)	○	○	○	○	○
Your child's school system (12)	○	○	○	○	○
Morehouse School of Medicine (13)	○	○	○	○	○
Emory University (14)	○	○	○	○	○

Q15 How much do you trust the United States Food and Drug Administration (FDA) to ensure the COVID-19 vaccine is safe for the public?

○ Fully trust (1)

○ Mostly trust (2)

○ Somewhat trust (3)

○ Do not trust (4)

Q16 How much do you trust the federal government to ensure a COVID-19 vaccine is safe for children?

- ○ Fully trust (1)
- ○ Mostly trust (2)
- ○ Somewhat trust (3)
- ○ Do not trust (4)

Q17 How easy has it been to get information about COVID-19 in your preferred language?

- ○ Very easy (1)
- ○ Somewhat easy (2)
- ○ Not easy (3)
- ○ Don't know (4)
- ○ Prefer not to answer (5)

---

*

Q18 What is your preferred language?

____

Q19 Overall, how confident do you feel using computers, smartphones, or other electronic devices to do the things you need to do online?

- ○ Very confident (1)
- ○ Somewhat confident (2)
- ○ Not too confident (3)
- ○ Not at all confident (4)
- ○ Prefer not to answer (5)

Q20 Please indicate which news outlets you trust for COVID-19 related news. This includes specific programming and podcasts published by these outlets

- ○ Network news (ABC, CBS, NBC, BBC) (1)
- ○ Breitbart (2)
- ○ Buzzfeed (3)
- ○ CNN (4)
- ○ Fox News (5)
- ○ HuffPost (6)
- ○ MSNBC (7)
- ○ New York Times (8)
- ○ Newsweek (9)
- ○ NPR (10)
- ○ PBS (11)
- ○ Politico (12)
- ○ The Guardian (13)
- ○ The Hill (14)
- ○ Time (15)
- ○ Univision (16)
- ○ USA Today (17)
- ○ Vice (18)
- ○ Vox (19)
- ○ Wall Street Journal (20)

- ○ Washington Post (21)
- ○ Local radio program (22)
- ○ Local cable TV program (23)
- ○ News broadcast from home country (via Internet, social media) (24)
- ○ None (25)
- ○ Prefer not to answer (26)

Q21
What is this a picture of?

____

Q22 **COVID-19 Testing and Disease Control**

Have you ever been tested for COVID-19?

- ○ Yes (1)
- ○ No (2)

Q23 How many times did you get the COVID-19 test?

- One (1)
- Two (2)
- Three (3)
- Four or more (4)
- Weekly (5)
- Monthly (6)

Q24 Are you required to test regularly for work or school?

- No (1)
- Yes, weekly (2)
- Yes, monthly (3)
- Yes, some other frequency (4)

Q25 Thinking of your most recent COVID-19 test, how easy was it to get the COVID-19 test?

- Very easy (1)
- Easy (2)
- Hard (3)
- Very Hard (4)

Q26 Why have you not been tested for COVID-19? Select all that apply.

- ☐ I haven't felt sick. (1)
- ☐ I felt sick, but I didn't feel sick enough to get tested. (2)
- ☐ I felt sick, but my health care provider told me to just stay home and away from other people. (3)
- ☐ I was told that testing wasn't available. (4)
- ☐ I didn't have a way to get to the testing location. (5)
- ☐ I didn't have the money to pay for a test. (6)
- ☐ I didn't know where to go to get tested. (7)
- ☐ I didn't have someone to watch my children or other people in my care so I could go get tested. (8)
- ☐ I couldn't take time off work to get tested. (9)
- ☐ I'm afraid that a positive test result will mean that I have to miss work. (10)
- ☐ I'm afraid to get a COVID-19 test. (11)
- ☐ I don't trust researchers. (12)
- ☐ I don't trust the government. (13)
- ☐ Other reason: __________ (14) ____

Q27 Have you ever tested positive for COVID-19?

- ○ Yes (1)
- ○ No (2)
- ○ I do not know or prefer not to answer (3)

Q28 Have you used an at-home test for COVID-19?

- ○ Yes (1)
- ○ No (2)

Q29 Please select answer choice "C" for this question.

- ○ A (1)
- ○ B (2)
- ○ C (3)
- ○ D (4)

Q30 **COVID-19 Vaccination**

Have you received at least one dose of the COVID-19 vaccine?

- ○ Yes, I got one-dose vaccine (Johnson & Johnson) (1)
- ○ Yes, I got first dose of two-dose vaccine (Moderna or Pfizer) (2)
- ○ Yes, I got both doses of two-dose vaccine (Moderna or Pfizer) (3)
- ○ No, I have not gotten the vaccine (4)
- ○ Don't know (5)
- ○ Prefer not to answer (6)

Q31 How likely are you to get a COVID-19 vaccine in the next 3 months?

- 0 (0)
- 1 (1)
- 2 (2)
- 3 (3)
- 4 (4)
- 5 (5)
- 6 (6)
- 7 (7)
- 8 (8)
- 9 (9)
- 10 (10)

Q32 What are the reasons why you have not yet gotten a COVID-19 vaccine? (Select all that apply.)

- ▢ I don’t like needles. (1)
- ▢ I don’t think I am at risk. (2)
- ▢ I don’t think vaccines work very well. (3)
- ▢ I don’t trust that the vaccine will be safe. (4)
- ▢ I’m concerned about side effects from the vaccine. (5)
- ▢ People in my family or community do not approve of the vaccine. (6)
- ▢ I already had COVID-19 so I do not think I need to get the vaccine. (7)
- ▢ It conflicts with my religious beliefs. (8)
- ▢ I am worried about being asked to show my ID at a vaccine appointment. (9)
- ▢ I am worried about being infected with COVID-19 by going to a vaccination location. (10)
- ▢ Other: ____ (11) ____

Q33 What has made it hard for you to get a COVID-19 vaccine? (Select all that apply.)

- ☐ I can't pay for it. (1)
- ☐ I don't know where to get vaccinated. (2)
- ☐ I don't have transportation to get there. (3)
- ☐ I cannot take time off work to get the vaccine. (4)
- ☐ I don't know how to make an appointment for a vaccination. (5)
- ☐ I don't have someone to watch my children/other people in my care while I go. (6)
- ☐ They don't speak my language at the vaccination location. (7)
- ☐ I don't have a social security number or government issued ID to get the COVID-19 vaccine. (8)
- ☐ Other: ________ (9) ____

Q34 Was there anything that made it hard to get a COVID-19 vaccine?

- ▢ I didn't know how to get an appointment for my vaccination. (1)
- ▢ The appointment took too long. (2)
- ▢ I was worried about being asked to show my ID at a vaccine appointment. (3)
- ▢ I didn't have transportation to or from a vaccination location. (4)
- ▢ Vaccination locations were too far or hard to get to. (5)
- ▢ I didn't know where to go for my vaccination. (6)
- ▢ I didn't have someone to watch my children/other people in my care while I went. (7)
- ▢ I couldn't take time off work for my vaccination. (8)
- ▢ They didn't speak my language at the vaccination location. (9)
- ▢ I was not able to access information about the COVID-19 vaccine in my preferred language. (10)
- ▢ I don't trust that the vaccine will be safe. (11)
- ▢ I was worried about being infected with COVID-19 by going to a vaccination location. (12)
- ▢ I was concerned about side effects from the vaccine. (13)
- ▢ I don't think vaccines work very well. (14)
- ▢ I'm allergic to vaccines. (15)
- ▢ I don't like needles. (16)
- ▢ It conflicts with my religious beliefs. (17)

☐ People important to me did not approve of me getting the vaccine. (18)

☐ Other____ (19)
____

Q35 Was there anything that made it hard to get a COVID-19 vaccine?

- ☐ I didn’t know how to get an appointment for my vaccination. (1)
- ☐ The appointment took too long. (2)
- ☐ I was worried about being asked to show my ID at a vaccine appointment. (3)
- ☐ I didn’t have transportation to or from a vaccination location. (4)
- ☐ Vaccination locations were too far or hard to get to. (5)
- ☐ I didn’t know where to go for my vaccination. (6)
- ☐ I didn’t have someone to watch my children/other people in my care while I went. (7)
- ☐ I couldn’t take time off work for my vaccination. (8)
- ☐ They didn’t speak my language at the vaccination location. (9)
- ☐ I was not able to access information about the COVID-19 vaccine in my preferred language. (10)
- ☐ I don’t trust that the vaccine will be safe. (11)
- ☐ I was worried about being infected with COVID-19 by going to a vaccination location. (12)
- ☐ I was concerned about side effects from the vaccine. (13)
- ☐ I don’t think vaccines work very well. (14)
- ☐ I’m allergic to vaccines. (15)
- ☐ I don’t like needles. (16)
- ☐ It conflicts with my religious beliefs. (17)

☐ People important to me did not approve of me getting the vaccine. (18)

☐ Other____ (19)
____

Q36 Do you plan to get the second dose of the vaccine?

○ Yes (1)

○ No (2)

○ Not Sure (3)

Q37 Why are you not planning to get the second dose? (Select all that apply)

- ☐ I am concerned about getting side effects from the second dose. (1)
- ☐ I am not sure the vaccine is safe. (2)
- ☐ I am not sure the vaccine is effective. (3)
- ☐ I believe one dose protects me from COVID-19. (4)
- ☐ My family or friends are not supportive of me getting the second dose. (5)
- ☐ I don’t have the time. (6)
- ☐ I can’t get to the vaccination site. (7)
- ☐ I can’t get off from work. (8)
- ☐ I had trouble getting an appointment. (9)
- ☐ I missed my appointment. (10)
- ☐ I was not able to re-schedule the appointment for my second dose. (11)
- ☐ My second dose was cancelled (they ran out of doses of the brand I needed). (12)
- ☐ Other (please specify):__________ (13)
____

Q38 Why are you ‘not sure’ if you are going to get the second dose? (Select all that apply).

- ▢ I am concerned about getting side effects from the second dose. (1)
- ▢ I am not sure the vaccine is safe. (2)
- ▢ I am not sure the vaccine is effective. (3)
- ▢ I believe one dose protects me from COVID-19. (4)
- ▢ My family or friends are not supportive of me getting the second dose. (5)
- ▢ I don’t have the time. (6)
- ▢ I can’t get to the vaccination site. (7)
- ▢ I can’t get off from work. (8)
- ▢ I had trouble getting an appointment. (9)
- ▢ I missed my appointment. (10)
- ▢ I was not able to re-schedule the appointment for my second dose. (11)
- ▢ My second dose was cancelled (they ran out of doses of the brand I needed). (12)
- ▢ Other (please specify): ____________ (13) ____

Q39 What would make you decide to get the second dose? (Select all that apply)

- ▢ If it was easy and convenient. (1)
- ▢ If I could get paid time off from work. (2)
- ▢ If I had more information about why the second dose is needed. (3)
- ▢ If I believed it was safe. (4)
- ▢ If I believed it was effective. (5)
- ▢ If my family and friends supported it. (6)
- ▢ If I believed it was important, not just for my protection, but for the protection of others. (7)
- ▢ If being fully vaccinated was required by work, school, or other activities I am involved in. (8)
- ▢ If I needed to be fully vaccinated to spend time with the people I care about. (9)
- ▢ Other (please specify): ________________ (10) ____

Q40 Why would you (or did you) get a COVID-19 vaccine? Select all that apply.

- ☐ I want to keep my family safe. (1)
- ☐ I want to keep my community safe. (2)
- ☐ I want to keep myself safe. (3)
- ☐ I have a chronic health problem, like asthma or diabetes. (4)
- ☐ My doctor told me to get a COVID-19 vaccine. (5)
- ☐ I don't want to get seriously sick from COVID-19. (6)
- ☐ I want to feel safe around other people. (7)
- ☐ I believe life won't go back to normal until most people get a COVID-19 vaccine. (8)
- ☐ My community or family expects me to. (9)
- ☐ I would not get a COVID-19 vaccine. (10)
- ☐ Other: ________________ (11) ____

Q41 Are you the parent or guardian of a child under age 18 years old living in your household?

- ○ Yes (1)
- ○ No (2)
- ○ Don't know (3)
- ○ Prefer not to answer (4)

Q42 Whether or not your child (children) are currently eligible for a COVID-19 vaccine, select the response that best describes what you have done or what you would do:

- Child is already vaccinated (1)
- Get them vaccinated right away (2)
- Wait a while to see how it is working (3)
- Only get your child vaccinated if their school requires it (4)
- Definitely not get them vaccinated (5)
- Don’t know (6)

Q43 How confident are you that the COVID-19 vaccines currently available in the U.S. are safe?

- Very confident (1)
- Somewhat confident (2)
- Not too confident (3)
- Not at all confident (4)
- Don't know (5)
- Prefer not to answer (6)

Q44 Are you willing to get a COVID-19 booster, when one is recommended for you?

- Yes (1)
- No (2)
- I have already received a COVID-19 booster shot (3)
- I don’t know (4)

Q45 What color is this? color

- Pink (1)
- Yellow (2)
- Blue (3)
- Brown (4)

Q46 **Research Participation**

Have you ever enrolled in a COVID-19 clinical trial?

- Yes, I signed up for a clinical trial for a COVID-19 vaccine. (1)
- Yes, I signed up for a clinical trial for a COVID-19 treatment. (2)
- No, I have never signed up for a COVID-19 clinical trial. (3)

Q47 Below are sources of information about COVID-19 clinical trials. How much do you trust each of these sources to give correct information?

	A great deal (1)	A fair amount (2)	Not very much (3)	None at all (4)	No opinion (5)
The National Institutes of Health (NIH) (1)	○	○	○	○	○
U.S. Federal Government (2)	○	○	○	○	○
Your doctor or health care provider (3)	○	○	○	○	○
Your local health care clinic or hospital (4)	○	○	○	○	○
University hospitals (5)	○	○	○	○	○
Companies that make drugs for medical use (6)	○	○	○	○	○
People who do research (7)	○	○	○	○	○
Friends, family, and community leaders (8)	○	○	○	○	○

Q48 If you get COVID-19, how willing would you be to sign up for a clinical trial for a COVID-19 treatment?

- 0 (0)
- 1 (1)
- 2 (2)
- 3 (3)
- 4 (4)
- 5 (5)
- 6 (6)
- 7 (7)
- 8 (8)
- 9 (9)
- 10 (10)

Q49 **Prevention**

In the past 7 days, how often have you chosen to do each of the following to keep yourself and others safe from COVID-19? (Do not include things you were required to do, such as wear a mask while visiting a store. Select one response for each row.)

	All the time (1)	Very Often (2)	Some of the time (3)	Never (4)
Worn a face covering or mask **indoors** in public places (1)	○	○	○	○
Washed my hands with soap or used hand sanitizer several times per day (2)	○	○	○	○
Stayed at least 6 feet away from other people who are not in my household (3)	○	○	○	○
Avoided **outdoor** public places, gatherings, and crowds (4)	○	○	○	○
Avoided **outdoor** gathering with friends or family (5)	○	○	○	○
Avoided **indoor** public places (6)	○	○	○	○
Avoided gathering **indoors** with friends or family (7)	○	○	○	○

Avoided travel in a bus, train, airplane, taxi, or share-ride car (8)	○	○	○	○

Q50 During the past 12 months, have you had a flu vaccine (either a shot or a spray in your nose)?

- ○ Yes (1)
- ○ No (2)
- ○ Don't know (3)
- ○ Prefer not to answer (4)

Q51 **Information, Trust, Risk Perception**

Whether you personally do these things or not, how safe, or unsafe do you feel doing the following activities right now?

	Very Unsafe (1)	Somewhat Unsafe (2)	Somewhat safe (3)	Very safe (4)	Don't know (5)
Grocery shopping (1)	○	○	○	○	○
Attending gatherings of more than 10 people (2)	○	○	○	○	○
Going to the hospital or doctor (3)	○	○	○	○	○
Dining in at restaurants (4)	○	○	○	○	○
Visiting with relatives or friends in their home (5)	○	○	○	○	○
Playing on playground equipment (6)	○	○	○	○	○
Going outside to walk, hike, or exercise (7)	○	○	○	○	○
Working outside the home (8)	○	○	○	○	○
Going to events (like sporting events and concerts) (9)	○	○	○	○	○

Exercising in gyms and studios (10)	○	○	○	○	○

Q52 Would you support or oppose requiring people to be vaccinated against COVID-19 to do the following things:

	Support (1)	Oppose (2)	Don't Know (3)
Attend sporting events or concerts (1)	○	○	○
Fly on an airplane (2)	○	○	○
Take a bus, train, or other form of public transit (3)	○	○	○
Attend college classes in person (4)	○	○	○
Attend K-12 schools in person (for age-eligible students) (5)	○	○	○
Work outside the home (6)	○	○	○

Q53 **Healthcare and Social Determinants of Health**

Is there a place that you usually go to when you are sick?

- ○ Yes (1)
- ○ No (2)
- ○ Don't know (3)

Q54 What kind of place do you go most often for medical care?

- ○ Community clinic or health center (1)
- ○ Family doctor or primary care provider (2)
- ○ Hospital ER (3)
- ○ Urgent care clinic at a hospital (4)
- ○ Urgent care clinic **not** at a hospital (5)
- ○ Retail center (for example, in a drug store) (6)
- ○ Some other place (7)
- ○ There is no one place I go to most often for medical care (8)

Q55 About how long has it been since you last saw a doctor or other health care professional about your health?

- ◯ Never (1)
- ◯ Within the past 12 months/1 year (2)
- ◯ 1 to 2 years ago (3)
- ◯ 3 to 4 years ago (4)
- ◯ 5 to 9 years ago (5)
- ◯ 10 years ago, or more (6)

Q56 Do you have any kind of health insurance or health care plan?

- ◯ Yes (1)
- ◯ No (2)
- ◯ Don't Know (3)

Q57 Did you lose health coverage during the COVID-19 pandemic?

- ◯ Yes (1)
- ◯ No (2)

Q58 What is the primary kind of health insurance or health care plan that you have now?

- ▢ Private health insurance through a job or school (1)
- ▢ Insurance bought through a government exchange such as healthcare.gov (2)
- ▢ Insurance bought from a health plan or company (3)
- ▢ Medicare (4)
- ▢ Medi-Gap (5)
- ▢ Medicaid (6)
- ▢ CHIP or kid's state insurance (e.g., PeachCare) (7)
- ▢ Peach State Health Plan (8)
- ▢ Military health care (9)
- ▢ Indian Health Service (10)
- ▢ Other: ________________ (11)

- ▢ Don't Know (12)

Q59 The COVID-19 pandemic may cause challenges for some people, whether they get COVID-19 or not. In the past month, have you experienced any of the challenges below?

	No, this is not a challenge (1)	Yes, this is a **minor** challenge (2)	Yes, this is a **major** challenge (3)	Not Applicable (N/A) (4)
Getting the health care, I need (including for mental health) (1)	○	○	○	○
Having a place to live (2)	○	○	○	○
Getting enough food to eat (3)	○	○	○	○
Having clean water to drink (4)	○	○	○	○
Getting the medications, I need (5)	○	○	○	○
Getting to where I need to go (6)	○	○	○	○
Taking care of my children or other people in my care (7)	○	○	○	○

Q60 **Health Literacy**

How confident are you filling out medical forms by yourself?

- ○ Very confident (1)
- ○ Somewhat confident (2)
- ○ Not too confident (3)
- ○ Not at all confident (4)
- ○ Prefer not to answer (5)

Q61 How often do you need someone to help you **read** written information from your doctor or drug store?

- ○ Never (1)
- ○ Rarely (2)
- ○ Sometimes (3)
- ○ Often (4)
- ○ Always (5)

Q62 How often do you need someone to help you **understand** written information from your doctor or drug store?

- Never (1)
- Rarely (2)
- Sometimes (3)
- Often (4)
- Always (5)

Q63 How often do you have a hard time understanding medical information that is told to you?

- Always (1)
- Often (2)
- Sometimes (3)
- Occasionally (4)
- Never (5)

---

*

Q64

What is this a picture of?

____

---

*

Q65 **Demographics**

What month were you born?

____

Q66 What year were you born? (Please enter as YYYY)

____

Q67 What is your gender?

- ○ Man (1)
- ○ Woman (2)
- ○ Transgender Female or Trans Woman (3)
- ○ Transgender Male or Trans Male (4)
- ○ Nonbinary, Genderqueer, or Genderfluid (5)
- ○ I would describe my gender as: ________________ (6)
- ○ Prefer not to answer (7)

Q68 Which of the following best describes how you think of yourself?

- ○ Gay (1)
- ○ Lesbian (2)
- ○ Straight (that is, not gay, lesbian, or bisexual) (3)
- ○ Bisexual (4)
- ○ Other (5)
- ○ Prefer not to answer (6)

Q69 Are you of Hispanic or Latino origin?

- ○ No (1)
- ○ Yes (2)

Q70 If yes, which of the following best describes your Hispanic/Latino heritage?

- ☐ Cuban (1)
- ☐ Colombian (2)
- ☐ Dominican (3)
- ☐ Guatemalan (4)
- ☐ Honduran (5)
- ☐ Mexican or Mexican American or Chicano (6)
- ☐ Puerto Rican (7)
- ☐ Salvadoran (8)
- ☐ Other (Please specify): (for example, Ecuadorian, Nicaraguan, Peruvian, Spaniard, Venezuelan) ________________ (9) ____

Q71 Which of the following best describes your race? (Select all that apply.)

- ☐ American Indian or Alaska Native (1)
- ☐ Asian (2)
- ☐ Black or African American (3)
- ☐ Native Hawaiian/Pacific Islander (4)
- ☐ White (5)
- ☐ Prefer not to answer (6)

Q72 American Indian or Alaska Native. Please Specify

☐ Enrolled in a federally recognized tribe (Specify: ______________) (1) ____

☐ Enrolled in a state recognized tribe (Specify: __________________) (2) ____

☐ Eligible for enrollment, but I am not enrolled in my tribe (Specify: ____________) (3) ____

☐ Not enrolled, but I am a descendent of an American Indian or Alaska Native tribe (4)

☐ Not applicable, my Indigenous People/Community do not have tribal enrollment (5)

☐ Other answer not specified here (Specify: ___________________) (6) ____

Q73 Is your location:

○ Urban (1)

○ Rural (2)

○ On a Reservation (3)

○ Other (Please specify): ________________ (4) ____

Q74 Asian. Please Specify

- ▢ Asian Indian (1)
- ▢ Chinese (2)
- ▢ Filipino (3)
- ▢ Japanese (4)
- ▢ Korean (5)
- ▢ Pakistani (6)
- ▢ Thai (7)
- ▢ Vietnamese (8)
- ▢ Cambodian (9)
- ▢ Hmong (10)
- ▢ Other (Please specify): (for example, Bangladeshi, Bhutanese, Burmese, Indonesian, Laotian, Malaysian, Mongolian, Nepalese, Okinawan, Sri Lankan, Taiwanese) ________________________ (11) ____

Q75 Black or African American. Please Specify

- ☐ African American (1)
- ☐ Ethiopian (2)
- ☐ Haitian (3)
- ☐ Jamaican (4)
- ☐ Nigerian (5)
- ☐ Somalian (6)
- ☐ Ghanaian (7)
- ☐ Trinidadian and Tobagonian (8)
- ☐ Other (Please specify): (for example, Barbadian, Cape Verdean, South African) ________________ (9) ____

Q76 Native Hawaiian/Pacific Islander. Please Specify

- ▢ Chamorro (1)
- ▢ Fijian (2)
- ▢ Marshallese (3)
- ▢ Native Hawaiian (4)
- ▢ Palauan/Belauan (5)
- ▢ Samoan (6)
- ▢ Tongan (7)
- ▢ Chuukese (8)
- ▢ Pohnpeian (9)
- ▢ Other (Please specify): (for example, Carolinian, I-Kiribati, Kosraean, Mariana Islander, Papua New Guinean, Saipanese, Tahitian, Tokelauan, Yapese) ________________________ (10) ____

Q77 White. Please Specify

- ☐ English (1)
- ☐ French (2)
- ☐ German (3)
- ☐ Irish (4)
- ☐ Italian (5)
- ☐ Polish (6)
- ☐ Other (Please specify): (for example, Scottish, Norwegian, Dutch) ________________________________ (7) ____

Q78 What is the highest degree or level of school you have completed?

- ○ Less than high school (for example, middle or elementary school) (1)
- ○ Some high school (2)
- ○ High school graduate (3)
- ○ GED (4)
- ○ Some college – no degree (5)
- ○ Associate’s or technical degree (for example, AA or AS) (6)
- ○ Bachelor’s degree (for example, BA, BS, or AB) (7)
- ○ Graduate degree (for example, MA or PhD) (8)
- ○ Prefer not to answer (9)

Q79 How many people live in your household right now? Include yourself, any other adults, and any children. Please Type a numeric value (i.e. 0, 1, 2, 3, 4 etc).

○ Minors (younger than age 18) (1) ____

○ Adults (age 18 to 64) (2) ____

○ Adults (Adults (age 65 and older) (3) ____

Q80 Which best describes your current employment status (select all that apply)?

▢ Working for pay—part time (less than 40 hours a week) (1)

▢ Working for pay—full time (40 hours a week or more) (2)

▢ Working without pay (for example, as an intern) (3)

▢ On leave from work (4)

▢ Unemployed and looking for a job (5)

▢ Unemployed and NOT looking for a job (6)

▢ Retired from work (7)

▢ Staying at home, taking care of the home or of others (8)

▢ Not able to work because of a disability (9)

▢ Going to school (10)

▢ Other: ______ (11) ____

Q81 In 2020, what was your total household income before taxes?

- ◯ Less than $15,000 (1)
- ◯ $15,000-$19,999 (2)
- ◯ $20,000-$24,999 (3)
- ◯ $25,000--$34,999 (4)
- ◯ $35,000-$49,999 (5)
- ◯ $50,000-$74,999 (6)
- ◯ $75,000-$99,999 (7)
- ◯ $100,000 and above (8)
- ◯ Prefer not to answer (9)

Q82 Were you born in the U.S.?

- ◯ Yes (1)
- ◯ No (2)
- ◯ Prefer not to answer (3)

Q83 Do you speak a language other than English at home?

- ◯ Yes. If yes, what language(s) (1) ____
- ◯ No (2)
- ◯ Prefer not to answer (3)

JS

Q84 What season is the hottest?

- ○ Spring (1)
- ○ Winter (2)
- ○ Fall (3)
- ○ Summer (4)

Q85 Thank you for taking the time to complete this survey. Please proceed to the next page to provide your information to receive your incentive. Incentives will be disseminated in order of completion.

Page Break

Q86
If you wish to receive a $25 gift card as our thank you for your time for participating in this survey please include your name, email, and mailing address in the fields below. Please be sure you have entered your information correctly so that we may send the gift card to the correct email address. Please also provide a correct mailing address in the event the gift card needs to be mailed.

First Name

____

Q87 Last Name

____

Q88 Email Address

____

Q89 Street Address

____

Q90 City

____

Q91 County of residence

____

Q92 State

____

Q93 Zip Code

____

Q94 Please type your email address again below for confirmation.

____

Q95 THANK YOU FOR YOUR INTEREST IN GEORGIA CEAL. For more information, please contact GEORGIA CEAL at georgiaceal@msm.edu and or 404-752-1700.

Please click the arrow to submit survey.

End of Block: Default Question Block
