# Supplementary material for: Challenges and Lessons Learned in Managing Web-Based Survey Fraud for the Garnering Effective Outreach and Research in Georgia for Impact Alliance–Community Engagement Alliance Survey Administrations
Source: JMIR Public Health Surveill. 2024 Dec 24;10:e51786. doi: 10.2196/51786 (PMC11687484; doi:10.2196/51786)
Supplement: Checklist 1 [file publichealth-v10-e51786-s004.docx]

| **Checklist Item** | **Page number with details on GEORGIA CEAL Surveys I and II** |
| --- | --- |
| **Design** | |
| Survey design | The target population was Black/African American and/or Hispanic/Latino/a (Latinx) adult residents of specified counties in Georgia counties (see page 4). |
| **Institutional Review Board (IRB) approval and informed consent process** | |
| IRB approval | All study procedures were approved by the Morehouse School of Medicine Institutional Review Board (IRB) and completed in accordance with institutional guidelines (see page 5). |
| Informed consent | Respondents were first asked to complete a series of screening questions to determine eligibility before being directed to a detailed consent form. Those who gave informed consent were then asked to complete the survey (see page 4). |
| Data protection | Participant privacy and confidentiality were protected via adherence to a strict data handling protocol; survey data with personal identifiers were downloaded onto encrypted servers, de-identified using unique ID numbers and stored as password-protected files (see page 5). |
| Development and testing | GEORGIA CEAL surveys were jointly developed by the National CEAL Assessment and Evaluation Workgroup, which defined optional and core items reflecting important themes and social determinants, and the GEORGIA CEAL Community Coalition Board (CCB), which refined and culturally adapted survey items to ensure relevance to Georgia communities. To minimize participant burden, surveys were designed to be completed within 15-20 minutes (see page 4). |
| **Recruitment process and description of the sample having access to the questionnaire** | |
| Open survey versus closed survey | Respondents were first asked to complete a series of screening questions to determine eligibility before being directed to a detailed consent form. Those who gave informed consent were then directed to an open survey, to complete a series of questions on demographics, social determinants of health and COVID-19 beliefs and experiences (see page 4). |
| Contact mode | Initial contact with potential participants was made on the Internet via an email outlining the study purpose, eligibility criteria, and participation incentive was shared with CCB members following a discussion during the regular monthly CCB meeting. The email included a pre-written recruitment message, with content in English and Spanish, survey links and recruitment flyers (paper and electronic) for broad distribution within the CCB’s contact lists/listservs and social media networks (see page 4). |
| Advertising the survey | News of the survey was shared via email and recruitment flyers (paper and electronic) for broad distribution within the CCB’s contact lists/listservs and social media networks (see page 4 and Multimedia Appendices). |
| **Survey administration** | |
| Web/E-mail | Data were collected online via the Qualtrics survey platform, with in-person data collection at GEORGIA CEAL community events, using iPads (see page 4). |
| Context | Potential participants were informed of the survey through the GEORGIA CEAL Community Coalition Board (CCB) network whose membership spans diverse organizations across the state and then directed to the online Qualtrics survey page (see page 4). |
| Mandatory/voluntary | The survey was voluntary (see page 4). |
| Incentives | Eligible individuals who consented to and completed the survey received a $25 e-gift card (see page 5). |
| Time/Date | GEORGIA CEAL Survey I data were collected between April 2021 – June 2021 while GEORGIA CEAL Survey II data collection spanned November 2021 – January 2022 (see page 4). |
| Randomization of items or questionnaires | N/A |
| Adaptive questioning | Screening questions determined eligibility and access to the survey instrument while skip logic was used within the questionnaire to condition access to specific questions (see page 4). |
| Number of Items | GEORGIA CEAL Surveys I and II contained 86 and 138 main questions, respectively, in either a single-question or matrix format (see page 4). |
| Number of screens (pages) | N/A (skip logic conditioned access to specific questions/pages). |
| Completeness check | N/A (questions were required with “Don’t know” and/or “Prefer not to answer” options available to protect participant right to decline answering required questions). |
| Review step | Participants were not allowed to change their answers via a back button feature (see page 4). |
| **Response rates** | |
| Unique site visitor | N/A |
| View rate (Ratio of unique survey visitors/unique site visitors) | N/A |
| Participation rate (Ratio of unique visitors who agreed to participate/unique first survey page visitors) | N/A |
| Completion rate (Ratio of users who finished the survey/users who agreed to participate) | N/A |
| **Preventing multiple entries from the same individual** | |
| Cookies used | N/A |
| IP check | IP address data were collected and reviewed but ultimately not used as flags given concerns that surveys collected at in-person events or from persons working in the same office or building may share the same IP address while opt-out services, virtual private networks (VPNs) and other privacy services allow persons to mask their IP address and location (see page 11). |
| Log file analysis | Other techniques used to identify multiple entries included personal identifiers (e.g., names and email addresses), and survey metadata (e.g., time and date stamps, geolocation; see page 5-7). |
| Registration | N/A |
| **Analysis** | |
| Handling of incomplete questionnaires | N/A |
| Questionnaires submitted with an atypical timestamp | Unreasonable completion timeframes (i.e., less than 10 minutes) and batched responses (i.e., identical timestamps) were excluded from analysis (see page 6). |
| Statistical correction | N/A |

This checklist has been completed as outlined in Eysenbach G. Improving the quality of Web surveys: the Checklist for Reporting Results of Internet E-Surveys (CHERRIES). J Med Internet Res. 2004 Sep 29;6(3):e34. doi: 10.2196/jmir.6.3.e34. Erratum in: doi:10.2196/jmir.2042. PMID: 15471760; PMCID: PMC1550605.
